# ﻿On small huntsman spiders (Araneae, Philodromidae) occurring in Guizhou and Hubei provinces, China

**DOI:** 10.3897/zookeys.1240.149456

**Published:** 2025-06-09

**Authors:** Jianshuang Zhang, Chengwen Zhang, Yang Zhong

**Affiliations:** 1 School of Life Sciences, Guizhou Normal University, Guiyang, Guizhou, China Guizhou Normal University Guiyang China; 2 School of Nuclear Technology and Chemistry & Biology, Hubei University of Science and Technology, Xianning, Hubei, China Hubei University of Science and Technology Xianning China

**Keywords:** Biodiversity, *COI*, DNA barcoding, fauna, morphology, new record, new species, running crab spiders, taxonomy

## Abstract

Spiders of the family Philodromidae Thorell, 1869 from Guizhou and Hubei provinces, China are studied. A total of three genera and seven species are reported and illustrated, comprising *Sinodromuslanyue***sp. nov.** (newly recorded genus for Hubei), and all known species from both provinces: *Philodromusauricomus* L. Koch, 1878, *P.guiyang* Long & Yu, 2022, *P.subaureolus* Bösenberg & Strand, 1906, *P.paiki* Jang, Lee, Yoo & Kim, 2024 (the previously records of *P.spinitarsis* Simon, 1895 from Guizhou and Hubei are presumed to be misidentifications, and should belong to *P.paiki*), *P.rufus* Walckenaer, 1826 (new record for Hubei) and *Tibellusjaponicus* Efimik, 1999 (new record for Hubei). Detailed descriptions, diagnoses, and illustrations of *S.lanyue***sp. nov.** and *P.guiyang* are given, and the male of *P.guiyang* is diagnosed and described in English for the first time. The other five species are also re-illustrated. Their DNA barcodes were obtained for species delimitation, matching of sexes and future use.

## ﻿Introduction

The Philodromidae Thorell, 1869, known as “running crab spiders” or “small huntsman spiders”, is a medium-sized family, comprising 30 genera and 527 valid species distributed worldwide, of which eight genera and 64 species have been recorded from China (WSC 2025). Among the 64 species, three – *Apollophaneslujiani* Lin & Li, 2024, *Psellonusdawanqu* Lin & Li, 2024, and *Pulchellodromusmainlingensis* (Hu & Li, 1987) – are endemic to the Guangxi Zhuang Autonomous Region, Guangdong Province, and Tibet Autonomous Region respectively, and the remaining 61 species belong to five genera that are widely distributed across multiple provinces and regions in China. Most of these 61 species have been documented from the Tibetan Plateau in the western part of the country, with other regions remaining relatively underexplored (Li and Lin 2016; Wang et al. 2024).

A recent study on philodromids conducted by colleagues from Jinggangshan University and Hunan Normal University, based on over a decade of surveys in southern China, described a new genus, *Sinodromus* Yao & Liu, 2024, which includes two new species (Wang et al. 2024), suggesting that the diversity of philodromid spiders in many regions of China remains poorly understood. Hubei Province, as a representative region of central China, is characterised by a relatively poor representation of the family Philodromidae, with only two species clearly recorded to date: *Philodromusspinitarsis* Simon, 1895 and *P.subaureolus* Bösenberg & Strand, 1906 (Li and Lin 2016). However, this record may not be entirely accurate due to potential misidentifications and is likely to underestimate the true diversity of philodromid spiders in the province.

Similar to Hubei Province, Guizhou has also been poorly represented in terms of Philodromidae diversity for a long time, with only one species recorded prior to 2022: *Tibellusjaponicus* Efimik, 1999 (Chen et al. 2003). Recently, a pioneering study on *Philodromus* species from Guizhou was conducted by Long et al. (2022), who identified five species from Guiyang, the capital of Guizhou Province: one species was described as new (*P.guiyang* Long & Yu, 2022), while four others were reported as new records for Guizhou: *P.auricomus* L. Koch, 1878, *P.rufus* Walckenaer, 1826, *P.spinitarsis* and *P.subaureolus*. However, upon re-examination of the specimens, those previously identified as *P.spinitarsis* were found to be misidentified and should instead belong to *P.paiki* Jang, Lee, Yoo & Kim, 2024 (Jang et al. 2024a). Therefore, *P.spinitarsis* is not believed to occur in Guizhou.

**Figure 1.**
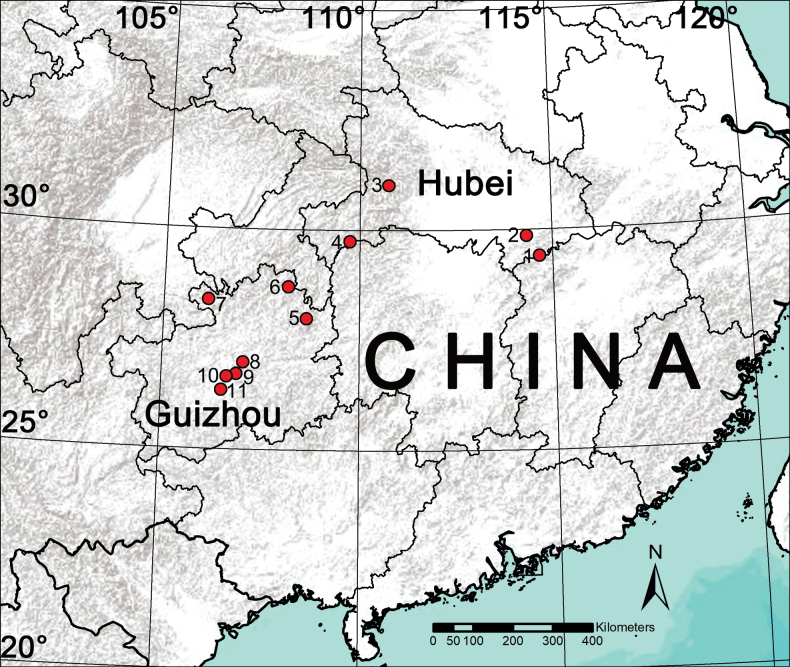
Sample collection locations for this project. Hubei Province **1** Jiugongshan National Nature Reserve **2** Hubei University of Science and Technology **3** Jiuling Mountain **4** Qizimei Mountain National Nature Reserve; Guizhou Province **5** Fanjingshan National Nature Reserve **6** Mayanghe National Nature Reserve **7** Xishui National Nature Reserve **8** Guiyang City, Kaiyang County **9** Guiyang City, Wudang District **10** Guiyang City, Yunyan District **11** Guiyang City, Huaxi District.

**Figure 2.**
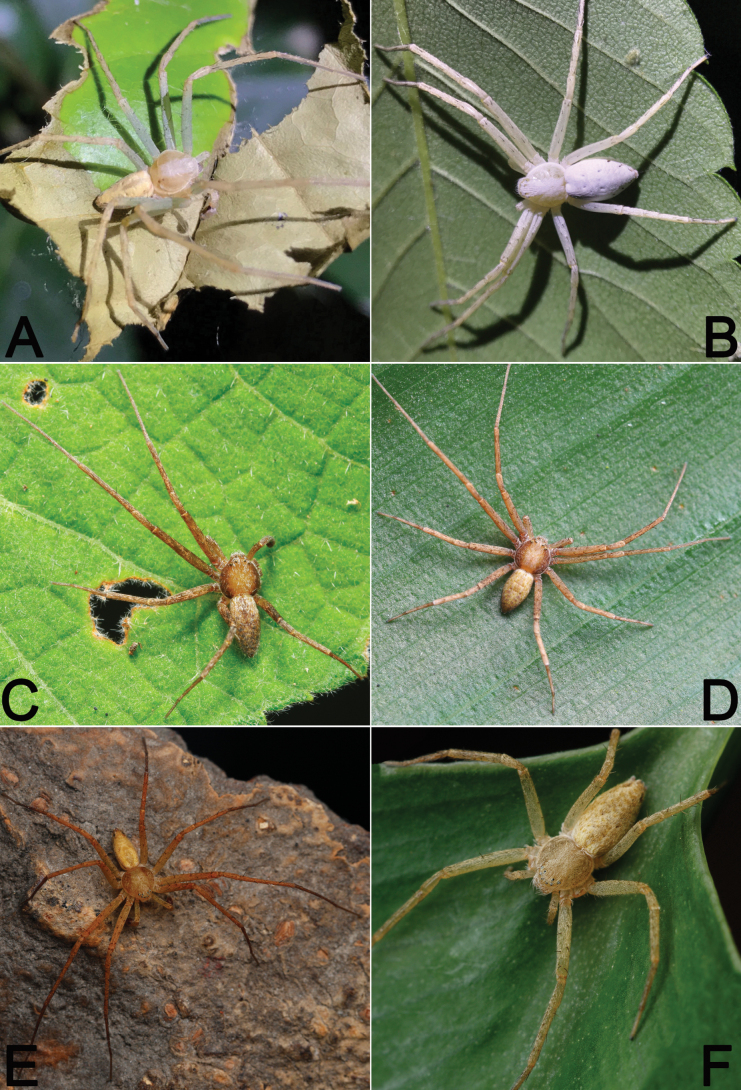
Living specimens of some Philodromidae species treated in this paper **A, B***Philodromusauricomus* (♂♀) **C, D***Philodromusguiyang* (♂) **E, F***Philodromussubaureolus* (♂♀). Photographs by Q. Lu (Shenzhen).

**Figure 3.**
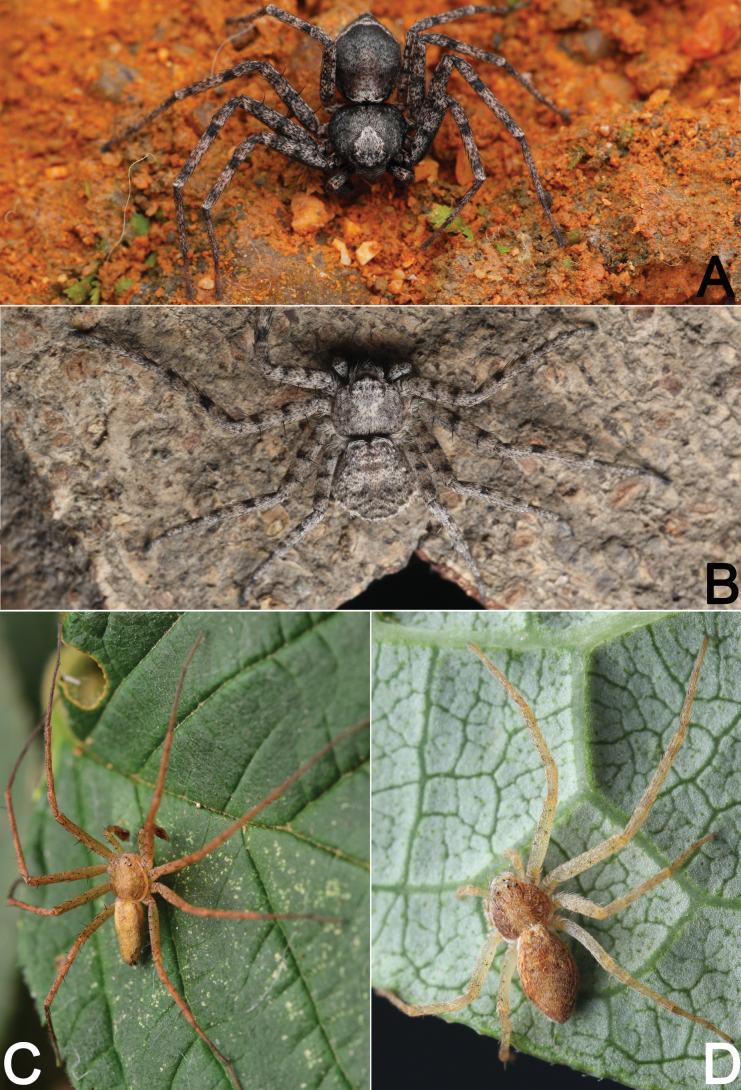
Living specimens of some Philodromidae species treated in this paper **A, B***Philodromuspaiki* (♂♀) **C, D***Philodromusrufus* (♂♀). Photographs by Q. Lu (Shenzhen).

**Figure 4.**
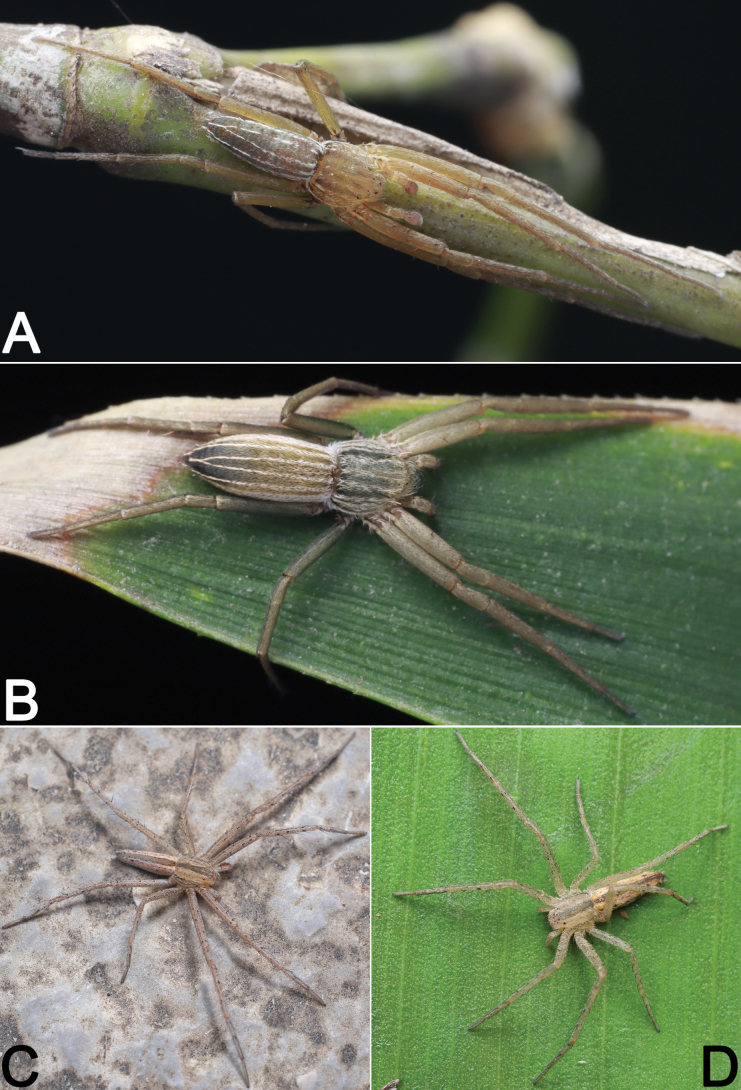
Living specimens of some Philodromidae species treated in this paper **A, B***Sinodromuslanyue* sp. nov. (♂♀) **C, D***Tibellusjaponicus* (♂♀). Photographs by Q. Lu (Shenzhen).

During the past decade, several field collections have been conducted in Hubei and Guizhou provinces by researchers from Hubei University of Science and Technology and Guizhou Normal University (Fig. 1). This paper reports our findings on the study of recently available samples from the area, which revealed seven philodromid species (Figs 2–4): one possesses certain characters associated with the genus *Sinodromus*, but can be easily distinguished from the other *Sinodromus* species; this species is new to science and is described under the name of *Sinodromuslanyue* sp. nov. The remaining six are identified as *P.auricomus*, *P.guiyang*, *P.subaureolus*, *P.paiki*, *P.rufus* (new record for Hubei), and *T.japonicus* (new record for Hubei).

The aim of the current paper is to present all species of philodromid spiders currently known from Hubei and Guizhou provinces, including: 1) description of *S.lanyue* sp. nov.; 2) diagnosis and description of the male of *P.guiyang* in English for the first time; 3) re-illustration of *P.auricomus*, *P.guiyang*, *P.subaureolus*, *P.paiki*, *P.rufus*, and *T.japonicus* based on new materials, including supplementary micrographs; and 4) DNA barcodes for all seven species, with the *COI* sequence barcodes of *S.lanyue* sp. nov., *P.auricomus*, *P.guiyang*, and *P.paiki* being published for the first time.

## ﻿Materials and methods

### ﻿Taxon sampling

Specimens in this study were collected alive by beating twigs and branches, and directly fixed in absolute ethanol, and then the right legs were removed to be stored at −80 °C for subsequent DNA extraction. The remainder of the specimens were preserved in 80% ethanol for identification and morphological examination. Besides newly available materials, all materials in Long et al. (2022), including the holotype of *P.guiyang* were re-examined for comparison.

For each species, we selected at least one male and one female specimen for DNA extraction and DNA barcode sequencing (Table 1). All examined material and voucher specimens are deposited in the Museum of Guizhou Normal University (**MGNU**), Guiyang, China.

**Table 1.** Voucher specimen information.

Specimen code	Sex	Genus	Species	COI GenBank accession no.	Sequence length
YHGY452	♂	*Philodromus*	*P.auricomus*	PV557367	632 bp
YHGY490	♀	*Philodromus*	*P.auricomus*	PV557368	632 bp
MYHPHI001	♀	*Philodromus*	*P.guiyang*	PV557358	632 bp
YHGY213	♂	*Philodromus*	*P.guiyang*	PV557360	632 bp
YHGY253	♀	*Philodromus*	*P.subaureolus*	PV557361	632 bp
YHGY254	♂	*Philodromus*	*P.subaureolus*	PV557362	632 bp
YHGY325	♀	*Philodromus*	*P.paiki*	PV557366	632 bp
YHPHI005	♂	*Philodromus*	*P.paiki*	PV557371	632 bp
YHGY304	♀	*Philodromus*	*P.rufus*	PV557365	632 bp
YHGY510	♂	*Philodromus*	*P.rufus*	PV557369	632 bp
YHGY511	♀	*Philodromus*	*P.rufus*	PV557370	632 bp
YHPHI008	♂	*Sinodromus*	*S.lanyue* sp. nov.	PV557372	632 bp
YHPHI009	♀	*Sinodromus*	*S.lanyue* sp. nov.	PV557373	632 bp
YHGY301	♂	*Tibellus*	*T.japonicus*	PV557363	632 bp
YHGY303	♀	*Tibellus*	*T.japonicus*	PV557364	632 bp
YHGY014	♀	*Tibellus*	*T.japonicus*	PV557359	632 bp

### ﻿Molecular protocols

Total genomic DNA was extracted using the Cell & Tissue Genomic DNA Isolation Kit (Bioteke, Beijing, China), following the manufacturer’s protocols. Following the standard polymerase chain reaction (PCR) settings, cytochrome *c* oxidase subunit I (*COI*) is amplified using the primer pairs LCO1490/HCO2198 (Folmer et al. 1994). For additional information on extraction, amplification and sequencing procedures, see Wheeler et al. (2016). Sequences were trimmed to 632 bp. All sequences were analysed using BLAST and are deposited in GenBank. The accession numbers are provided in Table 1.

### ﻿Morphological protocols

Specimens were examined using an Olympus SZX7 stereomicroscope and further details were studied under an Olympus CX41 compound microscope. Left male palps were examined and illustrated after dissection. Epigynes were removed and cleared in a warm 10% potassium hydroxide (KOH) solution. The vulvae of *S.lanyue* sp. nov. and *P.guiyang* were imaged after being embedded in Arabic gum. Images were captured with a Canon EOS 70D digital camera (20.2 megapixels) mounted on an Olympus CX41 compound microscope and assembled using Helicon Focus v. 6.80 image stacking software (Khmelik et al. 2005). All measurements were obtained using an Olympus SZX7 stereomicroscope and are given in millimetres. Eye diameters were measured at the widest part. The total body length does not include the chelicerae or spinnerets. Leg lengths are given as total length (femur, patella + tibia, metatarsus, tarsus). All distribution maps were generated with ArcGIS v. 10.5 (ESRI Inc). The terminology used in the text and figure legends follows Wang et al. (2024), Kubcová (2004a, b), Muster and Thaler (2004), and Jang et al. (2023, 2024a, b).

Abbreviations used in the text are as follows: **A** = atrium; **ALE** = anterior lateral eyes; **AME** = anterior median eyes; **AME–ALE** = distance between AME and ALE; **AME–AME** = distance between AMEs; **aSDL** = ascending part of sperm duct loop; **cCD** = course of copulatory duct; **CD** = copulatory duct; **CH** = clypeal height; **CO** = copulatory opening; **Con** = conductor; **CP** = cymbial process; **Cy** = cymbium; **dRTA** = dorsal branch of RTA; **dSDL** = descending part of sperm duct loop; **DTA** = dorsal tibial apophysis; **EA** = epigynal arch; **EG** = epigynal groove; **Em** = embolus; **EmB** = embolic base; **EmT** = embolic tip; **ET** = epigynal tooth; **FD** = fertilisation duct; **GH** = glandular head; **GM** = glandular mound; **IA** = internal sclerotised arch; **IR** = intertegular retinaculum; **MOQ** = median ocular quadrangle; **MOQA** = MOQ anterior width; **MOQL** = MOQ length; **MOQP** = MOQ posterior width; **MS** = median septum; **pCon** = prolateral part of conductor; **PLE** = posterior lateral eyes; **PME** = posterior median eyes; **PME–PLE** = distance between PME and PLE; **PME–PME** = distance between PMEs; **R** = receptaculum; **rMS** = rim of median septum; **rSEF** = rim of sclerotised epigynal fold; **RTA** = retrolateral tibial apophysis; **RTP** = retrolateral tegular projection; **SD** = sperm duct; **SDL** = sperm duct loop; **SEF** = sclerotised epigynal fold; **TA** = tegular apophysis; **tCon** = tip of conductor; **Te** = tegulum; **VPTA** = ventro-prolateral tibial apophysis; **vRTA** = ventral branch of RTA; **VTA** = ventral tibial apophysis.

## ﻿Results

### ﻿Taxonomic accounts

**Family Philodromidae Thorell, 1869**

#### ﻿Genus Philodromus

Taxon classificationAnimaliaAraneaePhilodromidae

Walckenaer, 1826

B7C1840A-FBB0-5934-84F1-0AE3D37D649E

##### Type species.

*Araneusaureolus* Clerck, 1757 from Europe, Turkey, Caucasus, Russia (Europe to Central Asia and Middle Siberia), Kazakhstan, Iran, Central Asia, Mongolia, China, Korea, Japan.

##### Diagnosis.

See Dondale and Redner (1976).

##### Comments.

*Philodromus* is the type genus of Philodromidae and currently includes 214 extant species that are found worldwide except for the Polar Regions (mainly distributed in the Old World and North America, except three from Australia and only one recorded in South America respectively) (WSC 2025), 21 species of which have been recorded from China. This genus is one of the largest of Philodromidae and comprises 41% of the total number of species of the family (WSC 2025).

Although *Philodromus* is rather well known for its high species diversity, the genus remains inadequately studied, and the species diversity is still insufficiently known (mostly related to its alpha taxonomy). The possible reasons include, but are not limited to the following: almost half of the species are described based on a single sex or juveniles (15 from males only, 79 from females only, eight from juveniles only) (WSC 2025); for many species described in earlier studies, original descriptions are rather brief, and illustrations are absent or inadequate (Long et al. 2022; WSC 2025); and the lack of available molecular data – we can obtain *COI* sequences for only 45 species through NCBI (2025).

The insufficiency of fundamental information in alpha taxonomy poses a significant obstacle to the advancement of beta taxonomy (i.e., phylogenetic studies). Several major taxonomic studies on a regional scale have been conducted, e.g., Dondale (1961, 1963), and Dondale and Redner (1968, 1969, 1975, 1976, 1978) for the North American species, Muster and his coauthors (Muster and Thaler 2004; Muster et al. 2007; Muster 2009), and Wunderlich (2012) for the European species; however, these revisionary studies often exclude species from Asia (potentially due to the aforementioned lack of foundational taxonomic information), and the debate on the group’s limits and internal structure of this family remains open (WSC 2025). According to the quite diverse copulatory structures of both sexes, different camouflage behaviours and habitat preferences, *Philodromus* sensu lato has been regarded as paraphyletic and needs to be split (Wunderlich 2012). However, we agree with Muster (2009) that the elevation of an autapomorphic species group would render *Philodromus* paraphyletic and in need of an extensive, large-scale review of the genus. Consequently, the present study follows the WSC (2025), and places all treated species in *Philodromus* sensu lato. We provide only the fundamental information on these species (such as detailed descriptions, supplementary illustrations, and DNA barcodes) for species delimitation, matching of sexes and future use. A review of the genus is not within the scope of this work.

#### ﻿ Philodromus auricomus

Taxon classificationAnimaliaAraneaePhilodromidae

L. Koch, 1878

3F824EFE-03AB-5367-BB99-7887435F2789

**Figure 5.**
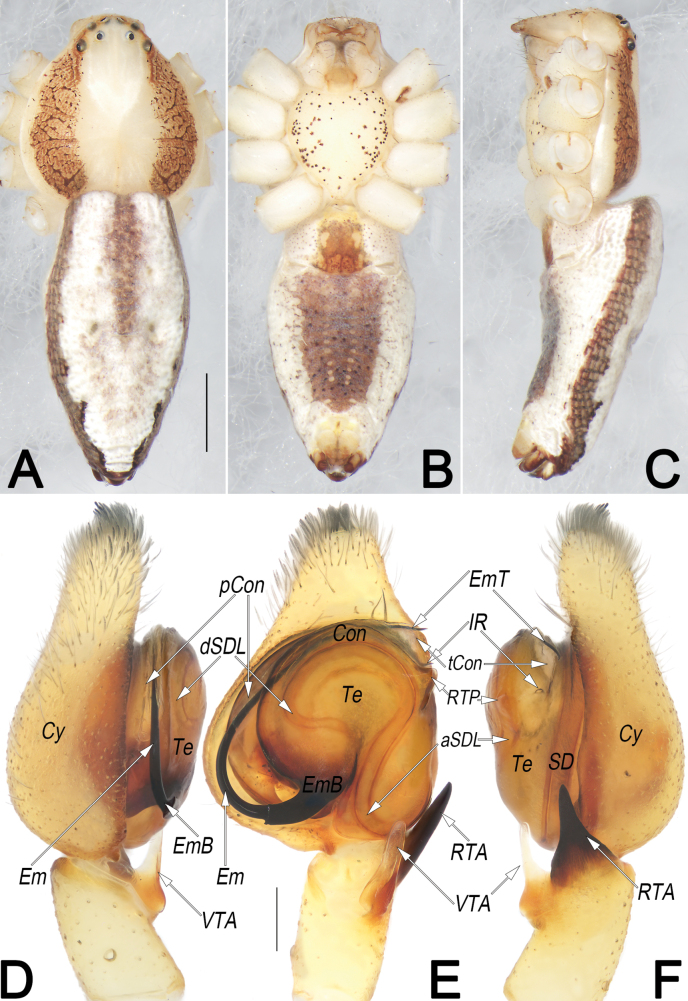
*Philodromusauricomus*, male, habitus (**A–C**) and left palp (**D–F**) **A** dorsal view **B** ventral view **C** lateral view **D** prolateral view **E** ventral view **F** retrolateral view. Abbreviations: aSDL = ascending part of sperm duct loop; Con = conductor; Cy = cymbium; dSDL = descending part of sperm duct loop; Em = embolus; EmB = embolic base; EmT = embolic tip; IR = intertegular retinaculum; pCon = prolateral part of conductor; RTA = retrolateral tibial apophysis; RTP = retrolateral tegular projection; SD = sperm duct; tCon = tip of conductor; Te = tegulum; VTA = ventral tibial apophysis. Scale bars: 1 mm (equal for **A–C**); 0.2 mm (equal for **D–F**).

**Figure 6.**
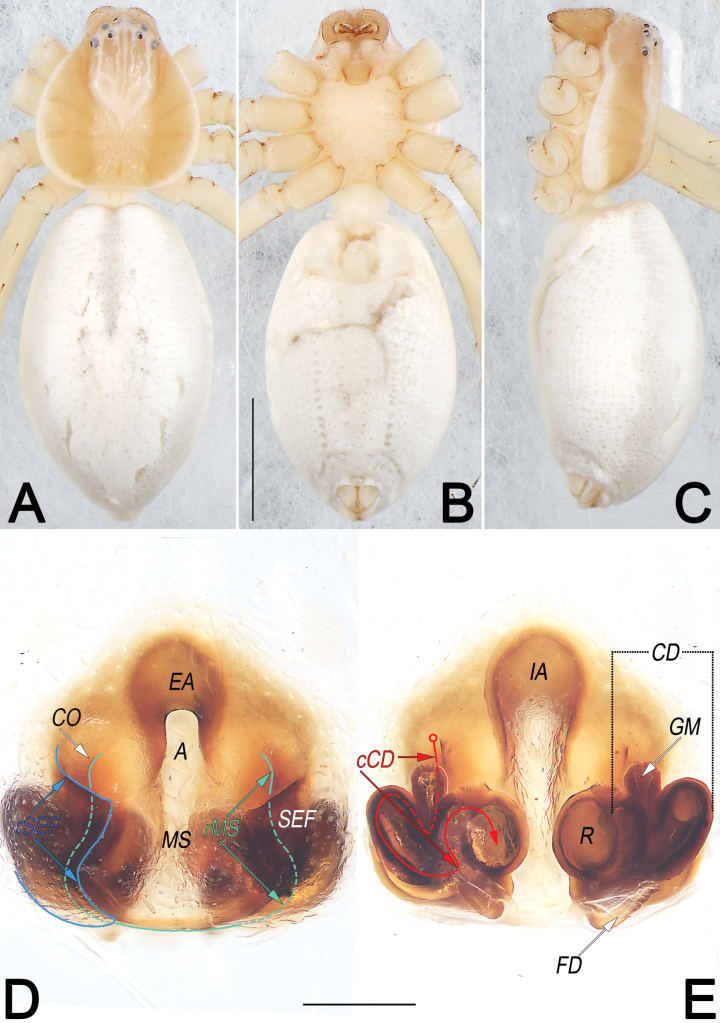
*Philodromusauricomus*, female, habitus (**A–C**) and epigyne (**D, E**) **A** dorsal view **B** ventral view **C** lateral view **D** ventral view **E** dorsal view. Abbreviations: A = atrium; cCD = course of copulatory duct (red line); CD = copulatory duct; CO = copulatory opening; EA = epigynal arch; FD = fertilisation duct; GM = glandular mound; IA = internal sclerotised arch; MS = median septum; R = receptaculum; rMS = rim of median septum (teal green line); rSEF = rim of sclerotised epigynal fold (blue line); SEF = sclerotised epigynal fold. Scale bars: 2 mm (equal for **A–C**); 0.2 mm (equal for **D, E**).

Figs 1 , 2A, B , 5 , 6 , 23A

Philodromus auricomus L. Koch, 1878: 763 (j); Yaginuma 1960: 101, fig. 86 (♀); Paik 1979: 425, figs 7–21 (♂♀); Zhang and Zhu 1982: 66, fig. 2b, d (♂); Ono 1988: 211 (S of Diaeasubadulta); Song and Zhu 1997: 183, fig. 127A–D (♂♀); Song et al. 1999: 470, figs 271C, 272C (♂♀); Yin et al. 2012: 1246, fig. 668a–e (♂♀); Kim and Lee (2017): 65, fig. 37A–E, pl. 13 (♂♀).
Diaea subadulta : Bösenberg and Strand1906: 258, pl. 13, fig. 302 (j).^1^

##### Material examined.

**China**: Guizhou Province: • 1♂, 1♀, Guiyang City, Huaxi District, Huaxi Wetland Park; 26.46°N, 106.67°E; 1140 m a.s.l.; 7 August 2022; H. Yu et al. leg. • 1♂, 1♀ (YHGY452 and YHGY490 used for sequencing, GenBank accession numbers in Table 1), Guiyang City, Huaxi District, University Town; 26.38°N, 106.65°E; 1173 m a.s.l.; 10 August 2024; Q. Jiang et al. leg. • 1♀, Tongren City, Fanjingshan National Nature Reserve, Yinjiang Tujia and Miao Autonomous County, Ziwei Town, Fengxiangping Village; 27.96°N, 108.58°E; 664 m a.s.l.; 19 June 2021; H. Yu et al. leg.

##### Diagnosis and description.

See Paik (1979), Song and Zhu (1997), Yin et al. (2012) and Kim and Lee (2017). Habitus as in Figs 5A–C, 6A–C. Male palp as in Fig. 5D–F, epigyne as in Fig. 6D, E.

##### Distribution.

Russia (Far East), China (Sichuang, Hebei, Shandong, Liaoning, Guizhou; distribution records in Guizhou as in Fig. 23A), Korea, Japan.

##### Comments.

The species was reported as a new record for Guizhou by Long et al. (2022) based on materials from Huaxi District in Guiyang City. New specimens examined indicate its distribution in the Fanjingshan Nature Reserve in Guizhou Province as well (Fig. 23A).

#### ﻿ Philodromus guiyang

Taxon classificationAnimaliaAraneaePhilodromidae

Long & Yu, 2022

5C66494B-BC5E-5EEA-A427-896890BAF57C

**Figure 7.**
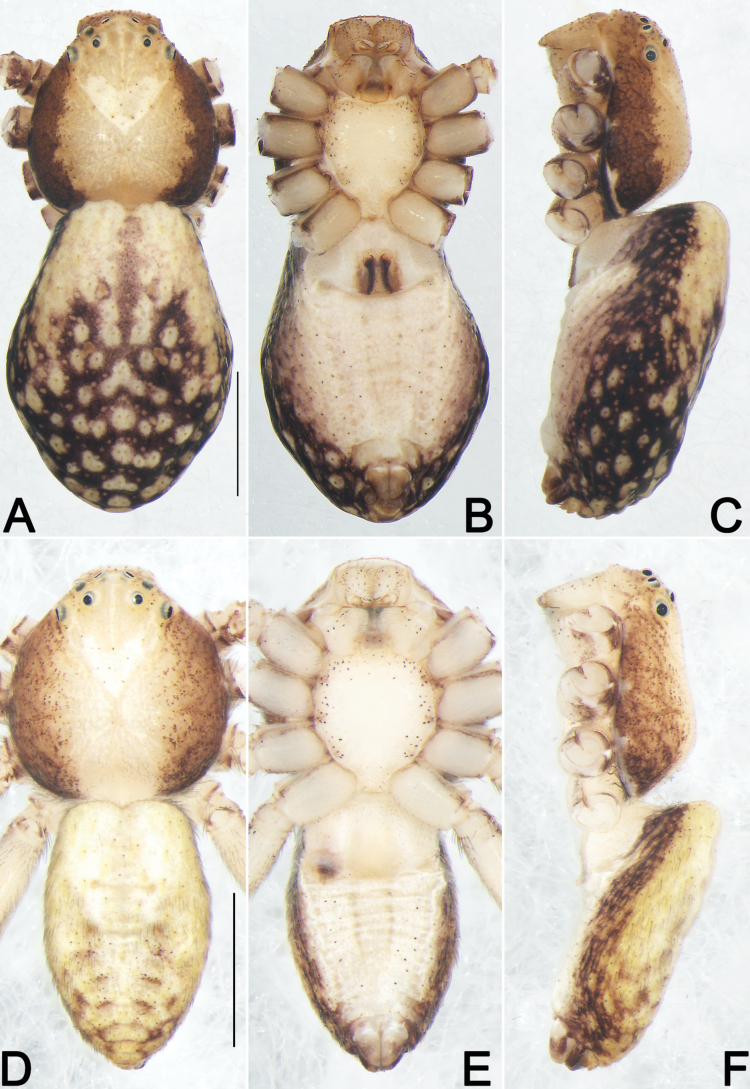
*Philodromusguiyang*, female (**A–C**) and male (**D–F**), habitus **A, D** dorsal view **B, E** ventral view **C, F** lateral view. Scale bars: 1 mm (equal for **A–C**, equal for **D–F**).

**Figure 8.**
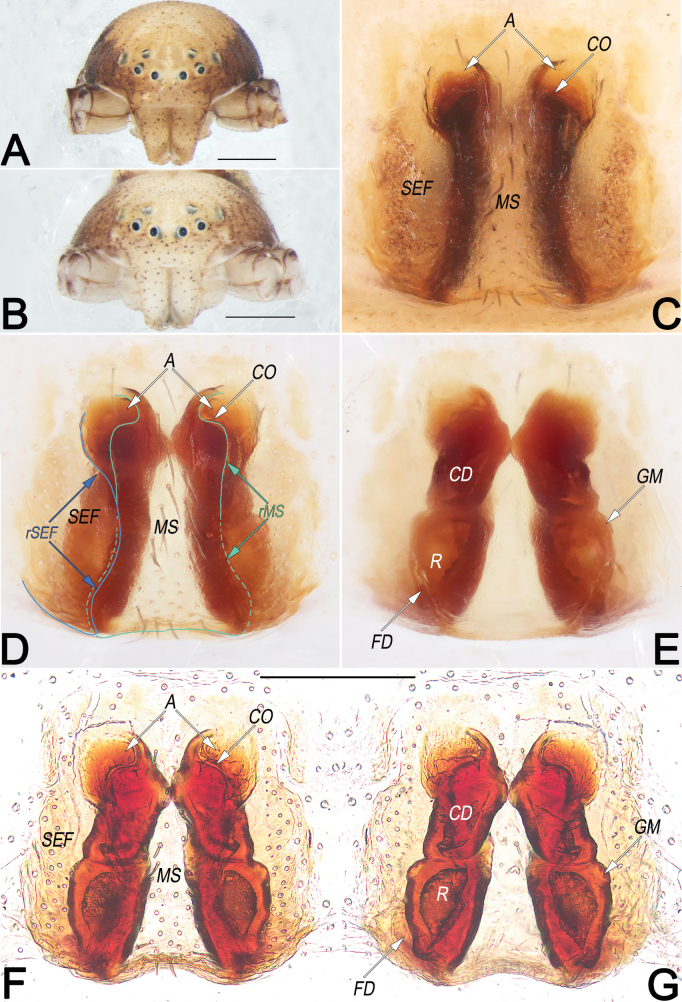
*Philodromusguiyang*, female (**A, C–G**) and male (**B**), frontal view of carapace (**A, B**) and epigyne (**C–G**) **A** female **B** male **C** intact, ventral view **D** macerated, ventral view **E** macerated, dorsal view **F** macerated and embedded in arabic gum, ventral view **G** macerated and embedded in Arabic gum, dorsal view. Abbreviations: A = atrium; CD = copulatory duct; CO = copulatory opening; FD = fertilisation duct; GM = glandular mound; MS = median septum; R = receptaculum; rMS = rim of median septum (teal green line); rSEF = rim of sclerotised epigynal fold (blue line); SEF = sclerotised epigynal fold. Scale bars: 0.5 mm (**A, B**); 0.2 mm (equal for **C–G**).

**Figure 9.**
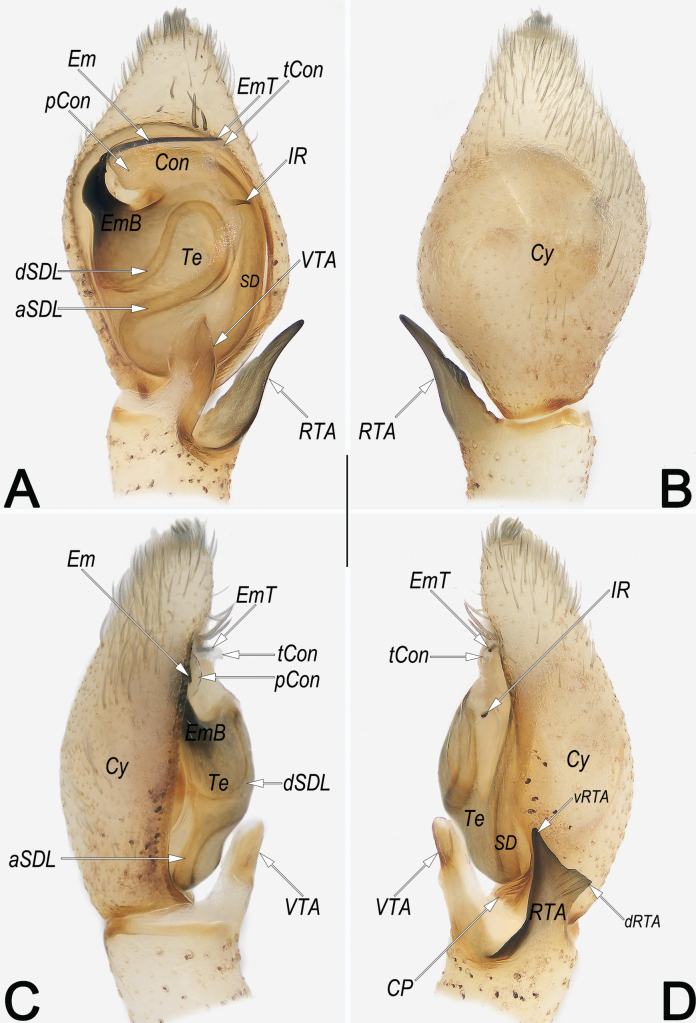
*Philodromusguiyang*, male, left palp **A** ventral view **B** dorsal view **C** prolateral view **D** retrolateral view. Abbreviations: aSDL = ascending part of sperm duct loop; Con = conductor; CP = cymbial process; Cy = cymbium; dRTA = dorsal branch of RTA; dSDL = descending part of sperm duct loop; Em = embolus; EmB = embolic base; EmT = embolic tip; IR = intertegular retinaculum; pCon = prolateral part of conductor; RTA = retrolateral tibial apophysis; SD = sperm duct; tCon = tip of conductor; Te = tegulum; vRTA = ventral branch of RTA; VTA = ventral tibial apophysis. Scale bar: 0.2 mm (equal for **A–D**).

Figs 1 , 2C, D , 7 , 8 , 9 , 23A

Philodromus guiyang Long & Yu, in Long et al. 2022: 118, figs 2A–D, 3A–D (♂); Wang et al. 2024: 281, figs 1A–D, 2A–E, 8A, 9A, B (♂♀).

##### Type material examined.

**China**: Guizhou Province: ***Holotype*** • ♂ (YHGY213 used for sequencing, GenBank accession numbers in Table 1), Guiyang City, Kaiyang County, Longgang Town, Pingshan Village, Zijiang Rift Scenic Area; 26.93°N, 107.07°E; 812 m a.s.l.; 10 June 2022; H. Yu & Q. Lu leg. ***Paratype*** • 1♂, the same data as the holotype.

##### Other material examined.

**China**: Guizhou Province: • 1♂, 1♀ (1♀, MYHPHI001 used for sequencing, GenBank accession numbers in Table 1), Tongren City, Mayanghe National Nature Reserve, Yanhe County, Huangtu Town; 28.69°N, 108.16°E; 1194 m a.s.l.; 8 August 2023; Y. Zhou et al. leg.

##### Diagnosis.

**Figure 10.**
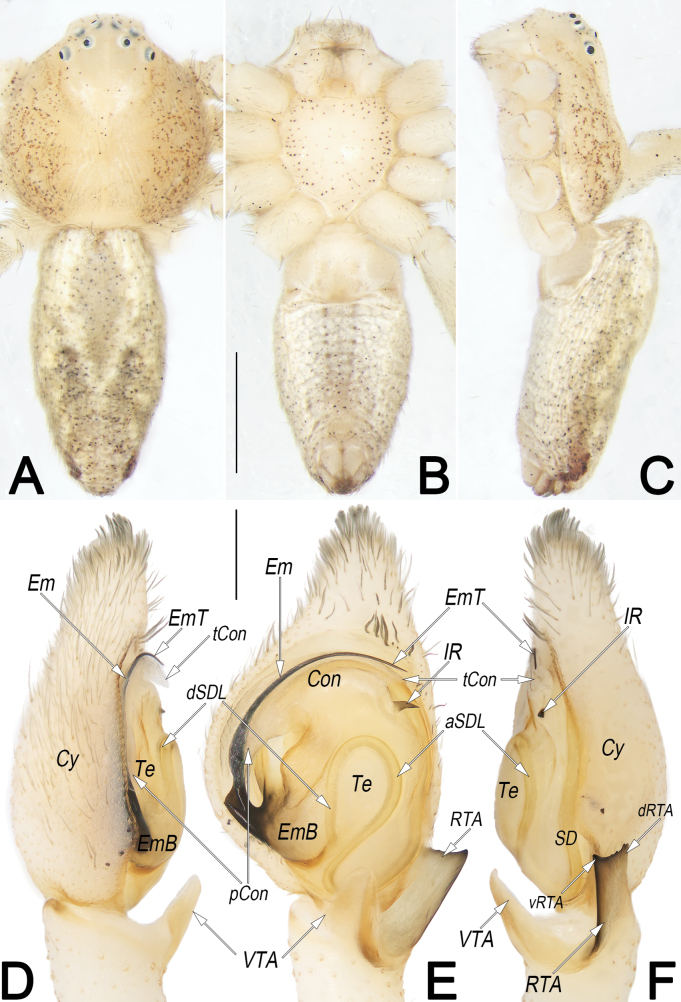
*Philodromussubaureolus*, male, habitus (**A–C**) and left palp (**D–F**) **A** dorsal view **B** ventral view **C** lateral view **D** prolateral view **E** ventral view **F** retrolateral view. Abbreviations: aSDL = ascending part of sperm duct loop; Con = conductor; Cy = cymbium; dRTA = dorsal branch of RTA; dSDL = descending part of sperm duct loop; Em = embolus; EmB = embolic base; EmT = embolic tip; IR = intertegular retinaculum; pCon = prolateral part of conductor; RTA = retrolateral tibial apophysis; SD = sperm duct; tCon = tip of conductor; Te = tegulum; vRTA = ventral branch of RTA; VTA = ventral tibial apophysis. Scale bars: 1 mm (equal for **A–C**); 0.2 mm (equal for **D–F**).

**Figure 11.**
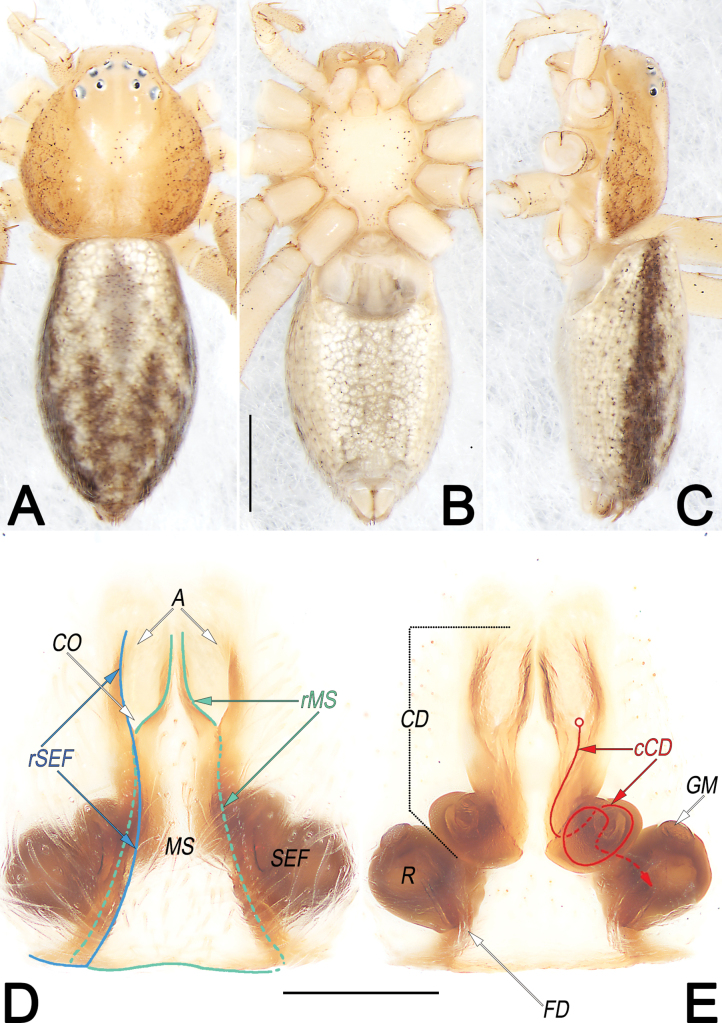
*Philodromussubaureolus*, female, habitus (**A–C**) and epigyne (**D, E**) **A** dorsal view **B** ventral view **C** lateral view **D** ventral view **E** dorsal view. Abbreviations: A = atrium; cCD = course of copulatory duct (red line); CD = copulatory duct; CO = copulatory opening; FD = fertilisation duct; GM = glandular mound; MS = median septum; R = receptaculum; rMS = rim of median septum (teal green line); rSEF = rim of sclerotised epigynal fold (blue line); SEF = sclerotised epigynal fold. Scale bars: 1 mm (equal for **A–C**); 0.2 mm (equal for **D, E**).

Females resemble those of *P.subaureolus* in having the similarly bell-shaped MS which is not delimited to SEF, but can be recognised by: (1) A comma-shaped (vs elongate-oval, nearly funnel-shaped) (cf. Fig. 8C, D, F and Fig. 11D); (2) anterior keel of MS relatively wider, ~ 1/5–1/4 epigyne width (vs distinctly narrower, ~ 1/10–1/8 epigyne width) (cf. Fig. 8D and Fig. 11D); (3) CD heavily sclerotised, not looped, distinctly thick and short, almost as thick as R, length ~ 1/2 epigyne (vs weakly sclerotised, distinctly thinner and longer, with a long cCD forming two loops before entering R, their thickness no more than 1/2 the diameter of R, length longer than epigyne) (cf. Fig. 8E, G and Fig. 11E); (4) R elongate-oval, anteriorly separated by ~ 0.5 × diameters, posteriorly separated by ~ 1.3 × diameters (vs nearly spherical, separated by ~1 diameter) (cf. Fig. 8E, G and Fig. 11E). Males of *P.guiyang* are also similar to those of *P.subaureolus* by the similar, blade-shaped VTA, lamellar RTA with a bifurcated tip, spine-shaped IR, and the more or less S-shaped SDL, but can be distinguished from the latter by: (1) Em claw-shaped, distinctly shorter, originating at the 10 o’clock position, terminating at the ~ 1 o’clock position (vs filiform, distinctly longer, originating at 8 o’clock position, terminating at ~ 1 o’clock position) (cf. Fig. 9A, C, D and Fig. 10D–F); (2) Con distinctly shorter, enveloping the second half of Em, the coverage ranges from the 11 o’clock to the 1 o’clock position (vs distinctly longer, almost enveloping the entire Em, the coverage ranges from the 9 o’clock to the 1 o’clock position) (cf. Fig. 9A, C, D and Fig. 10D–F); (3) in retrolateral view, the middle section of RTA distinctly narrower than its base and tip (vs not distinctly narrowed, the entire RTA is almost uniform in width) (cf. Fig. 9D and Fig. 10F); and (4) vRTA noticeably prominent, distinctly longer and sharper than dRTA (vs vRTA small, both branches of RTA are similar in size and shape) (cf. Fig. 9D and Fig. 10F).

##### Description.

**Female.** Total length 4.05. Carapace 1.62 long, 1.57 wide. Abdomen 2.65 long, 1.82 wide. ***Eye sizes and interdistances***: AME 0.07, ALE 0.07, PME 0.06, PLE 0.08, AME–AME 0.19, AME–ALE 0.10, PME–PME 0.36, PME–PLE 0.21, MOQL 0.31, MOQA 0.32, MOQP 0.48, CH 0.22. Sternum 0.89 long, 0.80 wide. ***Measurements of legs***: I 6.50 (1.85, 2.38, 1.40, 0.87), II 7.88 (2.24, 2.92, 1.71, 1.01), III 5.42 (1.72, 1.88, 1.20, 0.62), IV 5.57 (1.77, 1.91, 1.25, 0.64). Leg formula: II-I-IV-III. Cheliceral furrow with one promarginal tooth.

***Colouration in ethanol*** (Figs 7A–C, 8A). Carapace nearly pear-shaped, ocular region distinctly narrowed, tegument relatively smooth, with numerous hair bases (all hairs detached); lateral bands dark brown, ~ 1/6 of carapace width, respectively; median band wide, ~ 2/3 of carapace width, bright yellowish-brown and distinctly delimited to lateral bands, centrally with V-shaped white stripe starting from behind PLE, almost reaching indistinct cervical groove; radial furrows and fovea indistinctly marked. Cheliceral base coloured slightly darker than median band, with pale brown fangs. Sternum uniformly yellowish-white. Endites and labium coloured as cheliceral base, both with dense scopulae on anterior margins. Legs yellowish-brown, without distinct markings, and covered by short spines. Abdomen elongate-oval, dorsum with a narrow, purplish median band starting from behind pedicel, reaching 1/2 of abdomen length; posteriorly with numerous purplish-black streaks interspersed with many pale brown spots, forming a reticulated pattern; ventral abdomen uniformly pale brownish.

***Epigyne*** (Fig. 8C–G). Epigynal field slightly longer than wide; anterior and lateral margins not delimited, posterior margin rebordered; the arrangement of the various parts of the vulva (CD and R) are distinctly visible through integument. A small, located at anterior part of epigynal plate, divided by anterior keel of MS, represented by two comma-shaped cavities; the two cavities separated by ~ 1 diameter. MS bell-shaped, broad; anteriorly narrowed abruptly, ~ 1/5 epigyne width, with distinct edges and distinctly delimited to A; posteriorly widen gradually, ~ 1/2 epigyne width, with indistinct lateral rMS alongside with rSEF. SEF anteriorly and posteriorly narrowed, medially widened, ~ 1/3 epigyne width, not delimited to MS. CO indistinct, located at basolateral atrial borders, leading to CD which extend to connect with R. CD distinctly thick and heavily sclerotised, almost as thick as R, ~ 1/4 epigyne width; anteriorly convergent on the central axis, posteriorly descend obliquely, separated by ~ 1 diameter, finally connected to R at midlength of epigyne. R elongate-oval, ~ 1/2 epigyne length and 1/4 epigyne width, arranged obliquely; anteriorly separated by ~ 0.5 × diameter, posteriorly separated by ~ 1.3 × diameter. GM distinctly small, represented by small humps that locate at the antero-lateral surfaces of R. FD membranous and acicular, large, ~ 2/3 of R length, originating from the posterior surface of R, anterolaterally extending.

**Male.** Total length 3.25. Carapace 1.49 long, 1.42 wide. Abdomen 1.76 long, 1.15 wide. ***Eye sizes and interdistances***: AME 0.07, ALE 0.06, PME 0.06, PLE 0.08, AME–AME 0.16, AME–ALE 0.08, PME–PME 0.30, PME–PLE 0.19, MOQL 0.30, MOQA 0.30, MOQP 0.41, CH 0.19. Sternum 0.89 long, 0.81 wide. ***Measurements of legs***: I 7.78 (1.99, 2.66, 1.92, 1.21), II 9.70 (2.59, 3.27, 2.37, 1.47), III 6.05 (1.87, 2.01, 1.41, 0.76), IV 5.23 (1.79, 1.88, 1.03, 0.53). Leg formula: II-I-III-IV. Cheliceral furrow with one promarginal teeth. Colouration in ethanol as in females, but body slightly paler (Figs 7D–F, 8B; see Long et al. (2022) for others described).

***Palp*** (Fig. 9A–D). Tibia relatively long, ~ 2/3 of Cy length, with two apophyses arising distally from tibia. Both tibial apophyses are lamellar and almost equal in length, nearly as long as palpal tibia length, including: a weakly sclerotised VTA, blade-shaped in ventral view and finger-shaped in lateral views; and a more sclerotised RTA which with wide base, narrowed middle section, and more or less biforked tip; both branches of RTA nearly triangular, vRTA heavily sclerotised, surface and edges smooth, apex angle is approximately 30°, dRTA hyaline, surface rough and with several scratch-like textures, distal edge jagged, apex angle is approximately 90°. Cy distinctly longer than tibia, basoretrolaterally with an indistinct CP. Te oval, ~ 1.37 longer than wide, proximally slightly swollen, prolatero-apically slightly excavated to accommodate Em and Con. SD sinuate, originating at distal portion of Te, aligning clockwise along the tegular retrolateral margin, forming a S-shaped SDL in ventral view, finally terminating at the ~ 10 o’clock position, and entering EmB. Em claw-shaped, EmB thick, inserted prolatero-apically (approximately 10 o’clock relative to Te), gradually tapering toward apex; EmT sharp and retrolaterally pointed, terminating at ~ 1 o’clock position. Con membranous, axe-shaped, aligning transversely on apical part of the Te, enveloping the second half of Em. IR distinctly small, spine-shaped, located at the ~ 2 o’clock position.

##### Distribution.

China (Fujian, Guizhou, Hunan, Jiangxi; distribution records in Guizhou as in Fig. 23A).

##### Comments.

Long et al. (2022) described the holotype male of *P.guiyang* in Chinese in the original paper. Wang et al. (2024) later described the female of the species for the first time but did not provide a diagnosis and redescription for the male. Therefore, to date, the male of this species lacks an English description. Here we diagnose and describe the male in English for the first time. Newly available specimens indicate that the species is also distributed in Mayanghe National Nature Reserve in Guizhou Province (Fig. 23A).

#### ﻿ Philodromus subaureolus

Taxon classificationAnimaliaAraneaePhilodromidae

Bösenberg & Strand, 1906

ACE86CB8-D43D-5D55-BF8A-154AA2A266CC

Figs 1 , 2E, F , 10 , 11 , 23A, B

Philodromus subaureolus Bösenberg & Strand, 1906: 270, pl. 13, fig. 307 (♀); Braun 1965: 413, figs 95–99 (♂♀, S of P.aureolusjaponicola); Song et al. 1979: 19, fig. 13C (♂); Paik 1979: 439, figs 90–102 (♂♀); Song 1988: 134 (S of P.amitinus); Song and Zhu 1997: 197, fig. 140A–C (♂♀); Song et al. 1999: 477, figs 271N, 272I, 274A (♂♀); Yin et al. 2012: 1250, fig. 672a–d (♂♀); Kim and Lee 2017: 79, fig. 45A–D, pl. 16 (♂♀); Long et al. 2022: 118, figs 1G, H, 4A–F (♂).
Philodromus aureolus japonicola : Bösenberg and Strand 1906: 268, pl. 7, fig. 93, pl. 10, fig. 160 (♂♀); Yaginuma 1960: 102, fig. 87 (♀).
Philodromus amitinus : Chamberlin1924: 22, pl. 5, fig. 38 (♀).
Philodromus japonicola : Yaginuma 1962: 43 (elevated from subspecies of P.aureolus).^2^

##### Material examined.

**China**: Guizhou Province: • 1♀, Guiyang City, Huaxi District, Gao po Miao Town, Soupo Village; 26.26°N, 106.83°E, 1358 m a.s.l.; 21 May 2022; H. Yu et al. leg. • 1♂, Guiyang City, Huaxi District, Gao po Miao Town, Sanchahe Village; 26.27°N, 106.80°E; 1162 m a.s.l.; 20 May 2022; H. Yu et al. leg. • 1♂ (YHGY254 used for sequencing, GenBank accession numbers in Table 1), Guiyang City, Huaxi District, University Town; 26.38°N, 106.65°E; 1173 m a.s.l.; 9 June 2023; Q. Jiang et al. leg. • 1♂, Guiyang City, Yunyan District, Luchongguan Forest Park; 26.63°N, 106.70°E; 1310 m a.s.l.; 4 June 2022; H. Yu et al. leg. • 1♂, Guiyang City, Wudang District, Panlongshan Forest Park; 26.74°N, 106.88°E; 1172 m a.s.l.; 2 June 2022; H. Yu et al. leg. • 1♂, Guiyang City, Wudang District, Xinpu Buyi Town, Xiangzhigou Scenic Area, Guodijing canyon; 26.79°N, 106.91°E; 1059 m a.s.l.; 31 May 2022; H. Yu et al. leg. • 3♀, Guiyang City, Wudang District, Pianpo Buyi Town; 26.66°N, 106.93°E; 1308 m a.s.l.; 8 August 2021; H. Yu et al. leg. • 1♀, Guiyang City, Wudang District, Baoli Park; 26.63°N, 106.75°E; 1310 m a.s.l.; 5 June 2017; H. Yu et al. leg. • 3♂, 3♀ (1♀, YHGY253 used for sequencing, GenBank accession numbers in Table 1), Tongren City, Fanjingshan National Nature Reserve, Yinjiang Tujia and Miao Autonomous County, Guantai Mountain; 27.98°N, 108.69°E; 1025 m a.s.l.; 20 July 2021; H. Yu et al. leg.; Hubei Province: • 1♂, 1♀, Xianning City, Jiugongshan National Nature Reserve, , Yunzhonghu Scenic Area; 29.39°N, 114.65°E; 480 m a.s.l.; 3 July 2020; Q. Lu et al. leg. • 1♂, 1♀, Enshi Tujia and Miao Autonomous Prefecture, Qizimeishan National Nature Reserve, Xuanen County, Shadaogou Town, Meijiaya Village; 29.71°N, 109.76°E; 1155 m a.s.l.; 12 August 2019; H. Yu et al. leg. • 1♀, Yichang City, Zigui County, Guizhou Town, Jiuling Mountain; 31.03°N, 110.81°E; 150 m a.s.l.; 12 June 2019; H. Yu et al. leg.

##### Diagnosis and description.

See Paik (1979), Song and Zhu (1997), Yin et al. (2012), and Kim and Lee (2017). Habitus as in Figs 10A–C, 11A–C. Male palp as in Fig. 10D–F, epigyne as in Fig. 11D, E.

##### Distribution.

Mongolia, China (Zhejiang, Chongqing, Jiangsu, Anhui, Hubei, Henan, Shanxi, Shaanxi, Hebei, Gansu, Ningxia, Xinjiang, Inner Mongolia, Shandong, Liaoning, Jilin, Heilongjiang, Guizhou; distribution records in Hubei and Guizhou in Fig. 23), Korea, Japan.

##### Comments.

The species was reported as a new record for Guizhou by Long et al. (2022) based on materials from several districts in Guiyang City. New specimens examined also indicate its distribution in the Fanjingshan Nature Reserve in Guizhou Province (Fig. 23A). Many earlier studies, such as Song and Zhu (1997), Song et al. (1997), and Song et al. (1999) et al., recorded that this species was distributed in Hubei Province. However, only Song et al. (1997) specify its distribution in Jiuling Mountain (located within the current Yichang City), while the other studies did not provide specific distribution points or coordinates. Our recent survey, which included a re-investigation of the Jiuling Mountain area, shows that this species is distributed in at least three locations in Hubei Province: Jiuling Mountain, Qizimei Mountain, and Jiugong Mountain (Fig. 23B). This species is widely widespread in China (occurring in several provinces across the country), so it is likely that there are more distribution points for this species within the province. This will require further investigation to clarify.

#### ﻿ Philodromus paiki

Taxon classificationAnimaliaAraneaePhilodromidae

Jang, Lee, Yoo & Kim, 2024

51A5EFBD-0EDD-54D4-979A-F4650B2C3157

**Figure 12.**
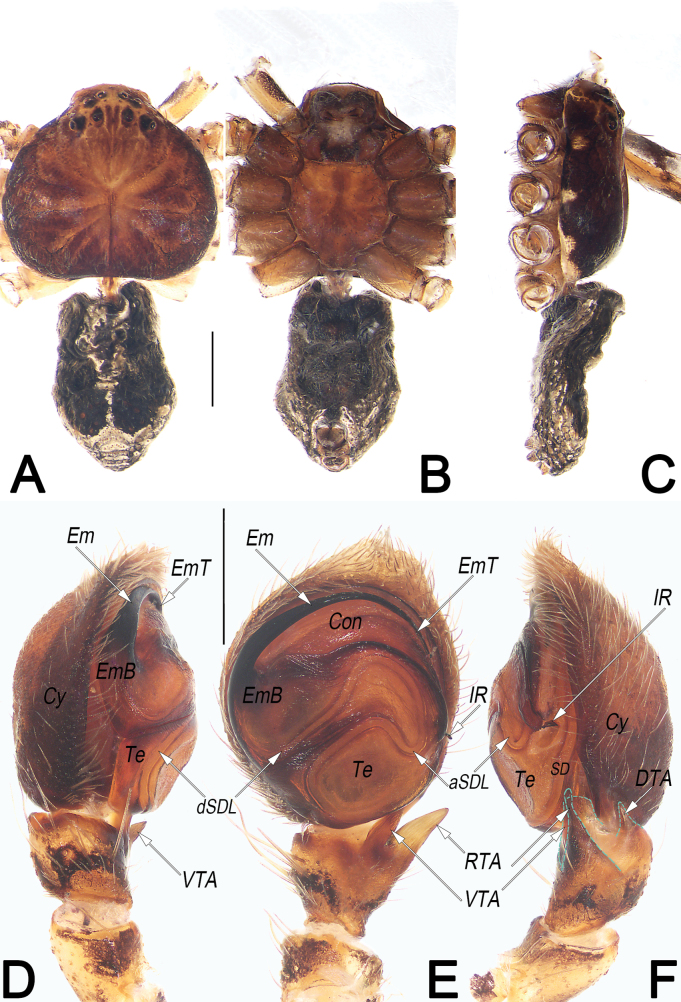
*Philodromuspaiki*, male, habitus (**A–C**) and left palp (**D–F**) **A** dorsal view **B** ventral view **C** lateral view **D** prolateral view **E** ventral view **F** retrolateral view (teal green dashed lines represent the contours of DTA, RTA and VTA). Abbreviations: aSDL = ascending part of sperm duct loop; Con = conductor; Cy = cymbium; dSDL = descending part of sperm duct loop; DTA = dorsal tibial apophysis; Em = embolus; EmB = embolic base; EmT = embolic tip; IR = intertegular retinaculum; RTA = retrolateral tibial apophysis; SD = sperm duct; Te = tegulum; VTA = ventral tibial apophysis. Scale bars: 1 mm (equal for **A–C**); 0.5 mm (equal for **D–F**).

**Figure 13.**
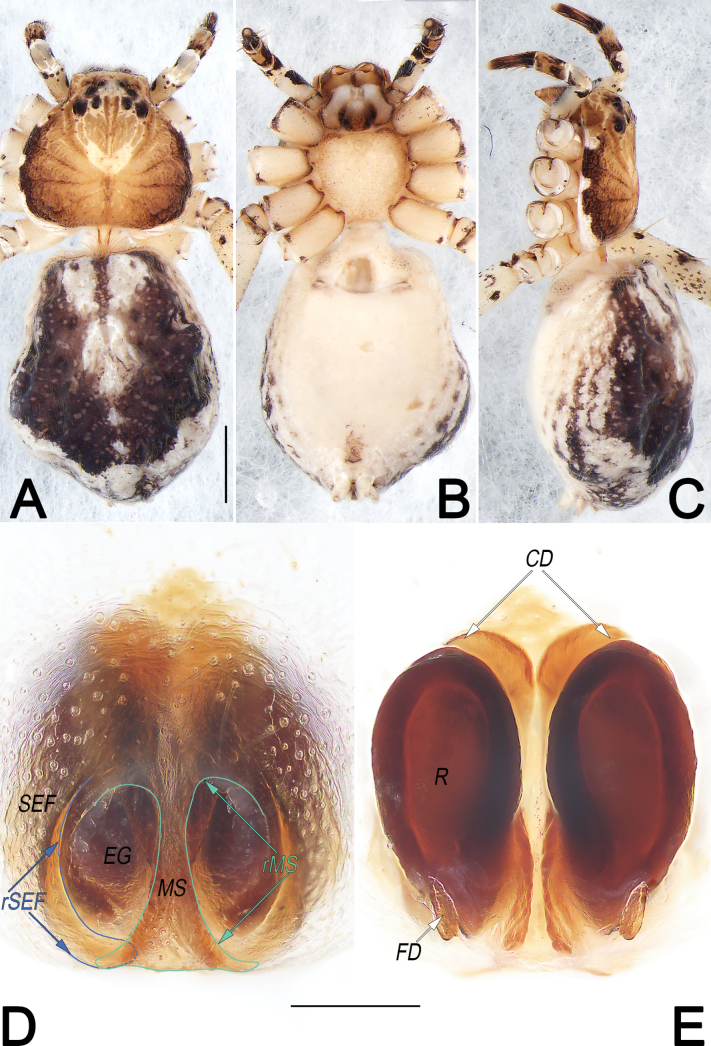
*Philodromuspaiki*, female, habitus (**A–C**) and epigyne (**D, E**) **A** dorsal view **B** ventral view **C** lateral view **D** ventral view **E** dorsal view. Abbreviations: CD = copulatory duct; EG = epigynal groove; FD = fertilisation duct; MS = median septum; R = receptaculum; rMS = rim of median septum (teal green line); rSEF = rim of sclerotised epigynal fold (blue line); SEF = sclerotised epigynal fold. Scale bars: 1 mm (equal for **A–C**); 0.2 mm (equal for **D, E**).

Figs 1 , 3A, B , 12 , 13 , 23A, B

Philodromus fuscomarginatus : Nakatsudi 1942: 14, fig. 5A, B (♂; misidentified per Jang et al. 2024a: 498); Paik 1979: 430, figs 40–60 (♂♀; misidentified per Jang et al. 2024a: 498).
Philodromus spinitarsis : Kim and Jung 2001: 201, figs 66–68 (♂♀; misidentified per Jang et al. 2024a: 498); Namkung 2002: 510, fig. 41.8a, b (♂♀; misidentified per Jang et al. 2024a: 498); Namkung 2003: 513, fig. 41.8a, b (♂♀; misidentified per Jang et al. 2024a: 498).
Philodromus poecilus : Kim and Lee 2017: 74, fig. 42A–D (♂♀; misidentified per Jang et al. 2024a: 498).
Philodromus paiki Jang et al. 2024a: 498, fig. 1A–K (♂♀).

##### Material examined.

**China**: Guizhou Province: • 1♂, 1♀, Guiyang City, Wudang District, Dongfeng Town, Guizhou Education University; 26.64°N, 106.80°E; 1071 m a.s.l.; 25 May 2015; H. Yu et al. leg. • 1♀ (YHGY325 used for sequencing, GenBank accession numbers in Table 1), Guiyang City, Kaiyang County, Longgang Town, Pingshan Village, Zijiang Rift Scenic Area; 26.93°N, 107.07°E; 812 m a.s.l.; 10 June 2022; H. Yu & Q. Lu leg.; Hubei Province: • 1♂, 1♀ (1♂, YHPHI005 used for sequencing, GenBank accession numbers in Table 1), Xianning City, Jiugongshan National Nature Reserve, Yunzhonghu Scenic Area; 29.39°N, 114.65°E; 480 m a.s.l.; 3 July 2020; Q. Lu et al. leg.

##### Diagnosis and description.

See Jang et al. (2024a). Habitus as in Figs 12A–C, 13A–C. Male palp as in Fig. 12D–F, epigyne as in Fig. 13D, E.

##### Distribution.

China (Guizhou, Hubei; Fig. 23), Korea.

##### Comments.

This species is easily confused with *P.spinitarsis* due to its similar genital morphology. Jang et al. (2024a) described it as a new species based on specimens from Korea and pointed out that previous records of *P.spinitarsis* in Korea in multiple studies were likely misidentifications, most of which should be attributed to *P.paiki*. It appears that many records of *P.spinitarsis* in Chinese literature may also be misidentifications. However, since we have not examined the original specimens, our study is currently based on materials from Guizhou and Hubei provinces.

Long et al. (2022) reported *P.spinitarsis* as a new record for Guizhou Province based on specimens from Guiyang City but did not provide diagnostic illustrations. Upon re-examining the specimens from Long et al. (2022), we determined that they should all belong to *P.paiki*. Therefore, there is currently no confirmed record of *P.spinitarsis* in Guizhou. The newly available specimens indicate that this species is also distributed in Fanjingshan National Nature Reserve, Guizhou Province (Fig. 23A).

Several studies have recorded the presence of *P.spinitarsis* in Hubei, such as Zhao (1993), Song and Zhu (1997), Song et al. (1999), Zhu and Zhang (2011), Yin et al. (2012), Zhang et al. (2022), but did not provide specific distribution points or coordinates. Additionally, the diagnostic illustrations provided are relatively crude and were not based on specimens from Hubei. Therefore, the reported distribution of *P.spinitarsis* in Hubei remains questionable. Our various field trips in Hubei have not yielded any *P.spinitarsis* specimens. In contrast, paired specimens of *P.paiki* were obtained from Jiugongshan National Nature Reserve (Fig. 23B). This paper represents the first formal report of the distribution of *P.paiki* in Hubei Province.

#### ﻿ Philodromus rufus

Taxon classificationAnimaliaAraneaePhilodromidae

Walckenaer, 1826

54DBB9BF-6D4B-5E9B-A1FD-0995D087E57A

**Figure 14.**
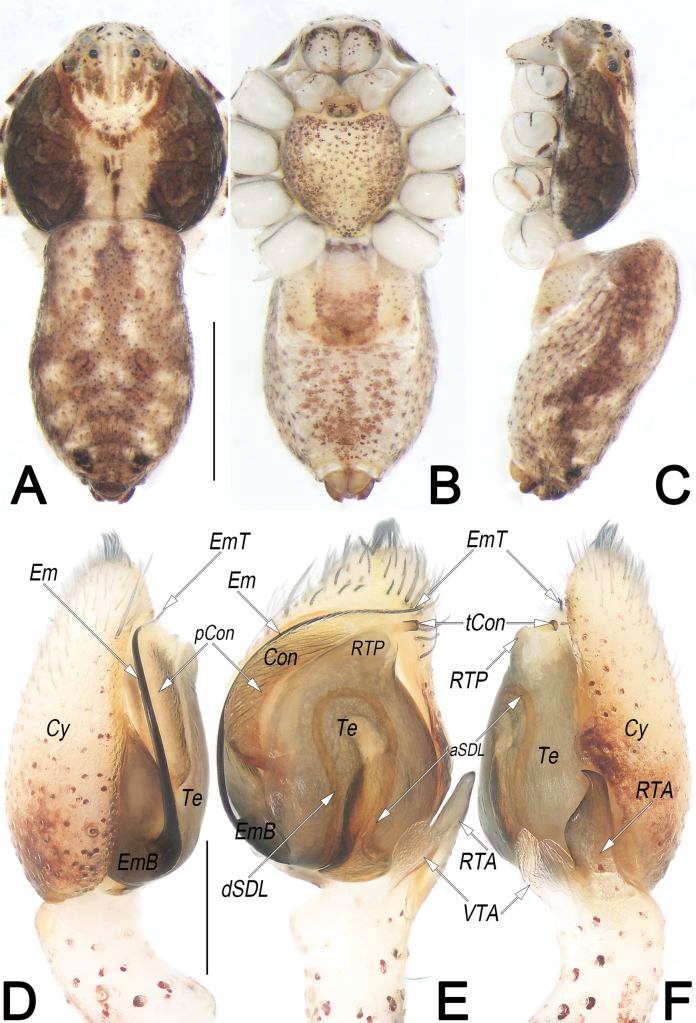
*Philodromusrufus*, male, habitus (**A–C**) and left palp (**D–F**) **A** dorsal view **B** ventral view **C** lateral view **D** prolateral view **E** ventral view **F** retrolateral view. Abbreviations: aSDL = ascending part of sperm duct loop; Con = conductor; Cy = cymbium; dSDL = descending part of sperm duct loop; Em = embolus; EmB = embolic base; EmT = embolic tip; pCon = prolateral part of conductor; RTA = retrolateral tibial apophysis; RTP = retrolateral tegular projection; tCon = tip of conductor; Te = tegulum; VTA = ventral tibial apophysis. Scale bars: 1 mm (equal for **A–C**); 0.2 mm (equal for **D–F**).

**Figure 15.**
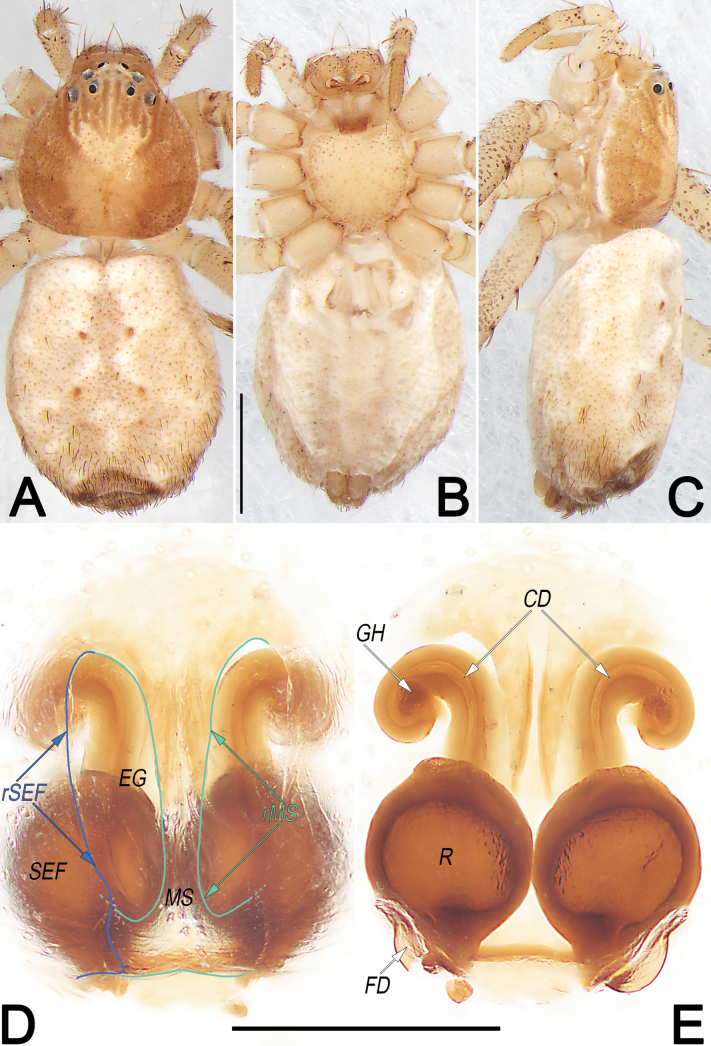
*Philodromusrufus*, female, habitus (**A–C**) and epigyne (**D, E**) **A** dorsal view **B** ventral view **C** lateral view **D** ventral view **E** dorsal view. Abbreviations: CD = copulatory duct; EG = epigynal groove; FD = fertilisation duct; GH = glandular head; MS = median septum; R = receptaculum; rMS = rim of median septum (teal green line); rSEF = rim of sclerotised epigynal fold (blue line); SEF = sclerotised epigynal fold. Scale bars: 1 mm (equal for **A–C**); 0.2 mm (equal for **D, E**).

Figs 1 , 3C, D , 14 , 15 , 23A, B

Philodromus rufus Walckenaer, 1826: 91; Walckenaer 1837: 555 (♂); Simon 1875: 287 (♀); Simon 1932: 854, 884, figs 1299, 1301 (♂♀); Zhu and Wang 1963: 477, fig. 28 (♀); Dondale 1964: 825, figs 1, 2, 5, 7–9 (♂♀, S of P.rufusvirescens); Yaginuma 1986: 216, fig. 121.3 (♀); Song et al. 1999: 476, fig. 271L (♀); Benjamin 2011: 19, fig. 62A–G (♂♀); Yin et al. 2012: 1248, fig. 670a, b (♀); Kim and Lee 2017: 76, fig. 43A–D, pl. 14 (♂♀); Zhang et al. 2022: 264, fig. 196A–G (♂♀); Jang et al. 2024b: 280, figs 1A–K, 2B, C (♂♀, S of P.pseudoexilis).
Philodromus clarkii : Blackwall 1850: 338 (♂);
Artama rufus : Simon 1864: 416.
Philodromus pellax : Herman 1879: 219, 371 (♂♀).
Philodromus clarae : Bertkau 1880: 246, pl. 6, fig. 1 (♂♀).
Philodromus pictus : Emerton 1892: 373, pl. 31, fig. 2 (♂♀); Emerton 1902: 37, figs 108–110 (♂♀).
Philodromus exilis : Banks 1892: 63, pl. 2, fig. 40 (♀).
Philodromus rufus virescens : Simon 1932: 854, 885 (♀).
Philodromus pseudoexilis : Paik 1979: 437, figs 81–89 (♂♀); Kim and Jung 2001: 199, figs 41–45 (♂♀).^3^

##### Material examined.

**China**: Guizhou Province: • 1♀, Guiyang City, Wudang District, Baoli Park; 26.63°N, 106.75°E; 1310 m a.s.l.; 5 June 2017; H. Yu et al. leg. • 1♂, 1♀ (YHGY510 and YHGY511 used for sequencing, GenBank accession numbers in Table 1), Guiyang City, Huaxi District, University Town; 26.38°N, 106.65°E; 1173 m a.s.l.; 15 July 2024; Q. Jiang et al. leg. • 1♂, 2♀ (1♀, YHGY304 used for sequencing, GenBank accession numbers in Table 1), Guiyang City, Huaxi District, Dangwu Town, Xiaba Village; 26.38°N, 106.60°E; 1138 m a.s.l.; 19 May 2022; H. Yu et al. leg. • 1♀, Guiyang City, Huaxi District, Gao po Miao Town, Diba Village; 26.26°N, 106.85°E; 1216 m a.s.l.; 21 May 2022; H. Yu et al. leg. • 1♀, Tongren City, Fanjingshan National Nature Reserve, Yinjiang Tujia and Miao Autonomous County, Guantai Mountain; 27.98°N, 108.69°E; 1025 m a.s.l.; 20 July 2021; H. Yu et al. leg.; Hubei Province: • 1♂, 1♀, Xianning City, Jiugongshan National Nature Reserve, Yunzhonghu Scenic Area; 29.39°N, 114.65°E; 480 m a.s.l.; 3 July 2020; Q. Lu et al. leg.

##### Diagnosis and description.

See Paik (1979), Song and Zhu (1997), Yin et al. (2012) and Jang et al. (2024b). Habitus as in Figs 14A–C, 15A–C. Male palp as in Fig. 14D–F, epigyne as in Fig. 15D, E.

##### Distribution.

North America, Europe, Turkey, Caucasus, Russia (Europe to Far East), Kazakhstan, Iran, Central Asia, Mongolia, China (Fujian, Yunnan, Sichuan, Xizang, Shanxi, Hebei, Gansu, Qinghai, Inner Mongolia, Liaoning, Jilin, Guizhou, Ningxia, Shandong, Shaanxi; distribution records in Guizhou and Hubei as in Fig. 23), Korea, Japan.

##### Comments.

This species is widely distributed and has been reported from several countries and regions across the Palearctic (WSC 2025). In China, the species has distribution records in multiple provinces, but there were no formal records of its presence in Guizhou Province before 2022, and no formal records in Hubei to date. Long et al. (2022) reported it as a new record for Guizhou based on specimens from Guiyang but did not provide diagnostic illustrations. Newly available specimens indicate that the species is also distributed in Fanjingshan National Nature Reserve in Guizhou Province and Jiugongshan National Nature Reserve in Hubei Province (Fig. 23). This paper represents the first formal report of the species’ distribution in Hubei Province.

#### ﻿Genus Sinodromus

Taxon classificationAnimaliaAraneaePhilodromidae

Yao & Liu, 2024

DA2C8499-E596-59E6-BFA4-15F38CA7339F

##### Type species.

*Sinodromusfujianensis* Yao & Liu, 2024 from Fujian and Jiangxi provinces, China.

##### Diagnosis.

See Wang et al. (2024).

#### ﻿ Sinodromus lanyue sp. nov.

Taxon classificationAnimaliaAraneaePhilodromidae

FAD04B00-75A1-5956-A2F3-9A3A4EBABB51

https://zoobank.org/FC654694-F8C2-46F2-9EC3-BD9EB53EE7EE

Figs 1 , 4A, B , 16 , 17 , 18 , 19 , 20 , 23B

##### Type material.

**China**: Hubei Province: ***Holotype*** • ♂: Xianning City, Xianan District, Hubei University of Science and Technology, the bamboo forest on the hill behind Lanyue Lake; 29.85°N, 114.34°E; 22 March 2023; Y. Zhong & Q. Lu leg. (Inventory number: MGNU-2025-PHISL001). ***Paratypes*** • 1♂, 1♀ (Inventory number: MGNU-2025-PHISL002~003), the same data as the holotype.

##### Other material examined.

1♂, 1♀ (YHPHI008 and YHPHI009 used for sequencing, GenBank accession numbers in Table 1), the same data as the holotype.

##### Etymology.

The species name is derived from the type locality; noun in apposition.

##### Diagnosis.

**Figure 16.**
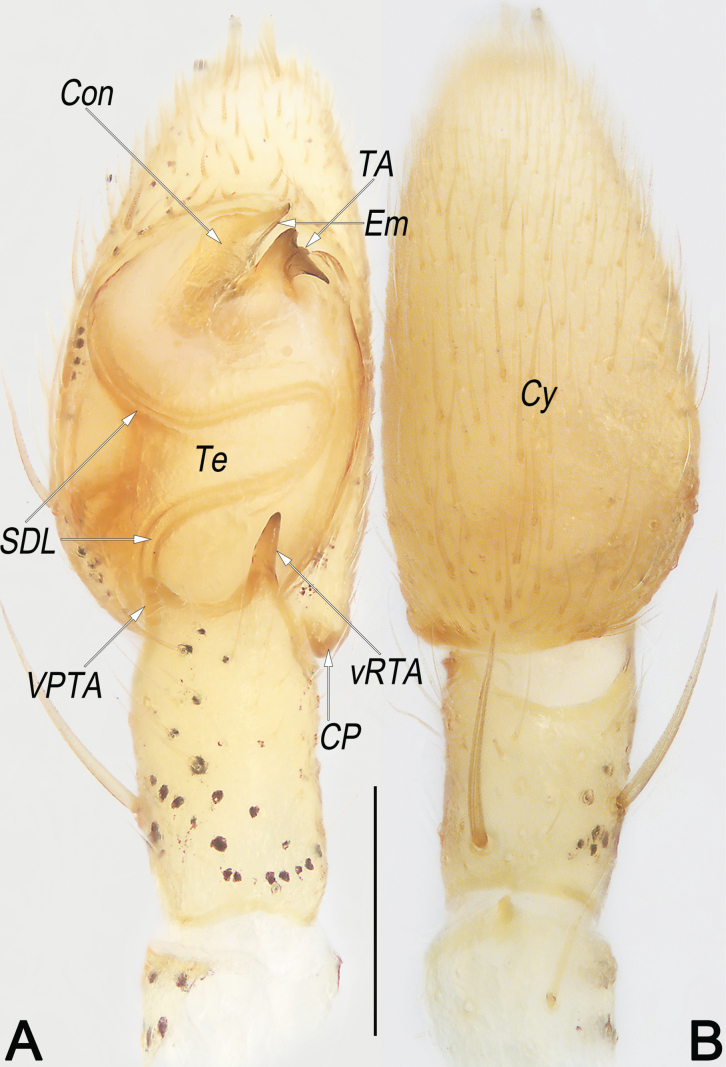
*Sinodromuslanyue* sp. nov., holotype male, left palp **A** ventral view **B** dorsal view. Abbreviations: Con = conductor; CP = cymbial process; Cy = cymbium; Em = embolus; SDL = sperm duct loop; TA = tegular apophysis; Te = tegulum; VPTA = ventro-prolateral tibial apophysis; vRTA = ventral branch of RTA. Scale bar: 0.2 mm (equal for **A, B**).

**Figure 17.**
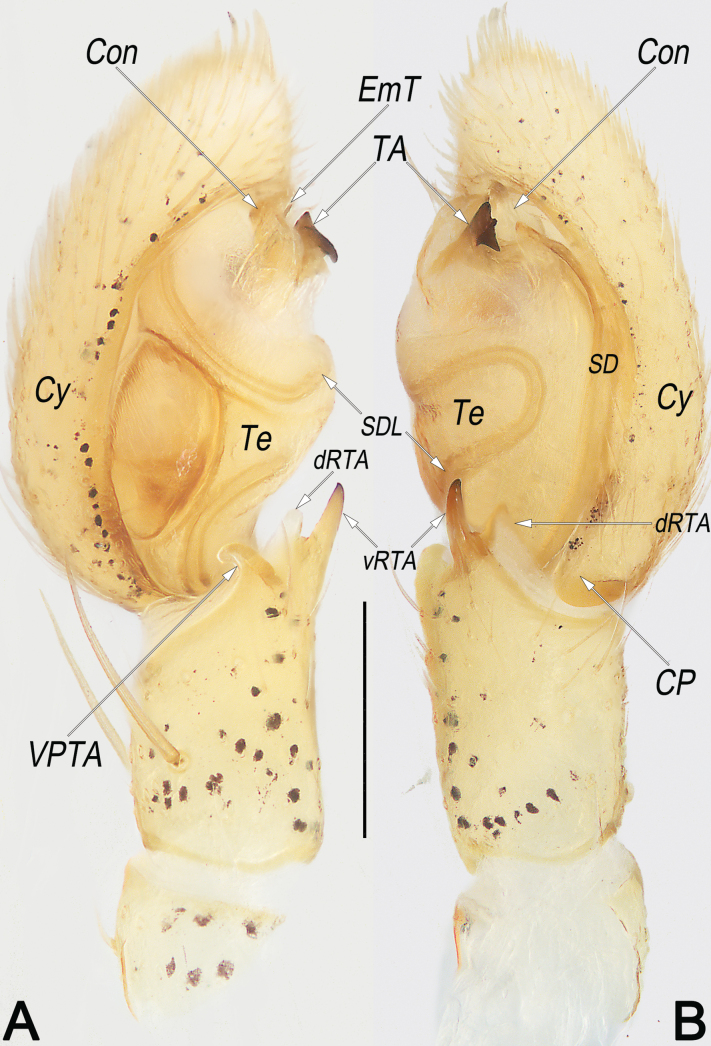
*Sinodromuslanyue* sp. nov., holotype male, left palp **A** prolateral view **B** retrolateral view. Abbreviations: Con = conductor; CP = cymbial process; Cy = cymbium; dRTA = dorsal branch of RTA; EmT = embolic tip; SD = sperm duct; SDL = sperm duct loop; TA = tegular apophysis; Te = tegulum; VPTA = ventro-prolateral tibial apophysis; vRTA = ventral branch of RTA. Scale bar: 0.2 mm (equal for **A, B**).

**Figure 18.**
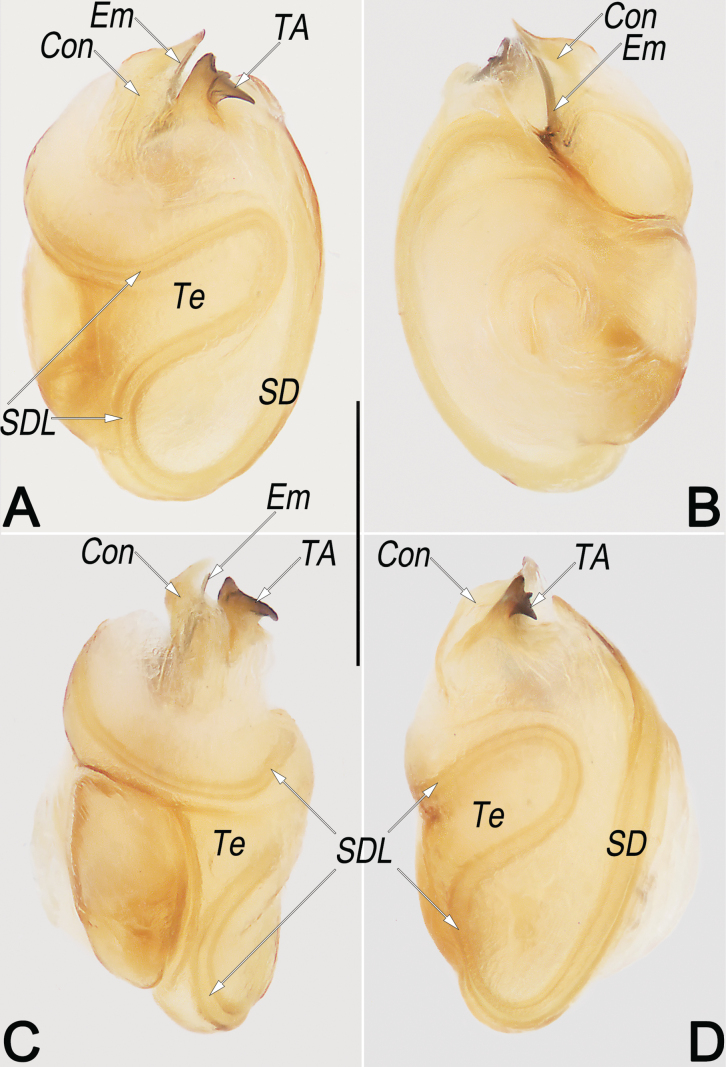
*Sinodromuslanyue* sp. nov., holotype male, left palpal bulb **A** ventral view **B** dorsal view **C** prolateral view **D** retrolateral view. Abbreviations: Con = conductor; Em = embolus; SD = sperm duct; SDL = sperm duct loop; TA = tegular apophysis; Te = tegulum. Scale bar: 0.2 mm (equal for **A–D**).

**Figure 19.**
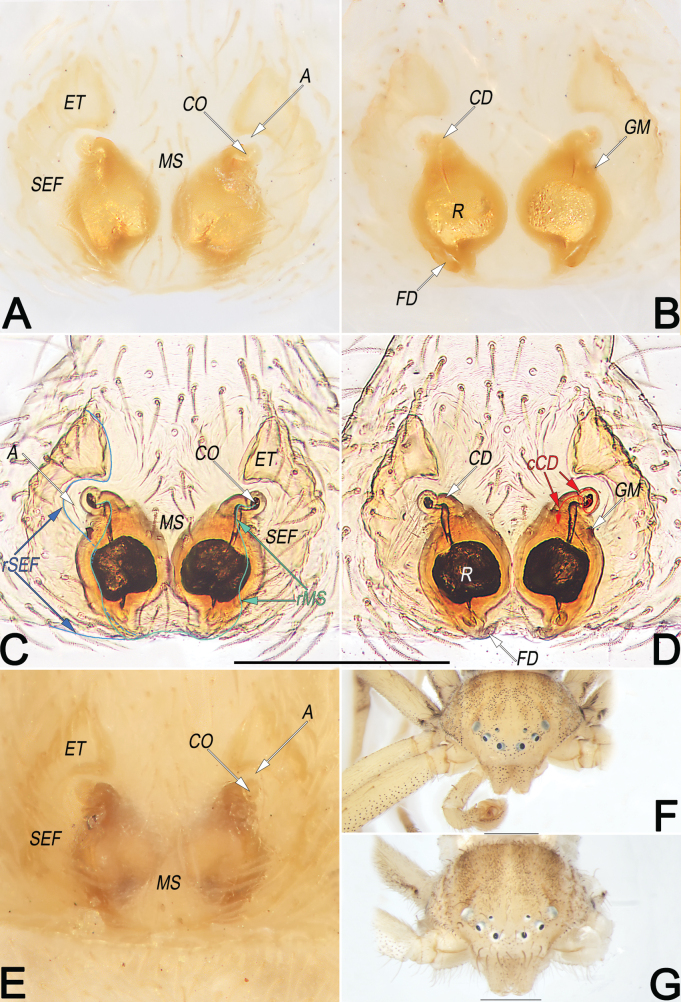
*Sinodromuslanyue* sp. nov., paratype female (**A–E, G**) and holotype male (**F**), epigyne (**A–E**) and frontal view of carapace (**F, G**) **A** macerated, ventral view **B** macerated, dorsal view **C** macerated and embedded in arabic gum, ventral view **D** macerated and embedded in arabic gum, dorsal view **E** intact, ventral view **F** male **G** female. Abbreviations: A = atrium; cCD = course of copulatory duct (red line); CD = copulatory duct; CO = copulatory opening; ET = epigynal tooth; FD = fertilisation duct; GM = glandular mound; MS = median septum; R = receptaculum; rMS = rim of median septum (teal green line); rSEF = rim of sclerotised epigynal fold (blue line); SEF = sclerotised epigynal fold. Scale bars: 0.2 mm (**A–E**); 0.5 mm (equal for **F, G**).

Males of the new species are easily distinguished from *Sinodromusfujianensis* Yao & Liu, 2024 (only congener with described male) by the following combination of morphological characteristics: (1) TA shaped like a cock’s head, with a hump-like, not folded basal apophysis (vs horn-shaped, with a lamellar, folded basal apophysis) (cf. Figs 16A, 17A, B, 18A, C, D and Wang et al. 2024: figs 4B–E, 5C, G, I, K) ; and (2) Con wider than TA, surface relatively smooth (vs narrower than TA, with many scaly serrations) (cf. Figs 16A, 17A, B, 18A–D and Wang et al. 2024: figs 4B–E, 5C, G, I, K). Females of the new species resemble those of *S.perbrevis* Yao & Liu, 2024 in having a similar MS and endogyne but can be recognised by: (1) ET axe-shaped, distinctly widened, wider than midsection of SEF (vs ear-shaped, not widened, nearly as wide as midsection of SEF) (cf. Fig. 19A, C, E and Wang et al. 2024: figs 7C, 8C); and (2) R oval, close together (vs globular, widely separated) (cf. Fig. 19B, D and Wang et al. 2024: fig. 7D).

##### Description.

**Male (MGNU-2025-PHISL001).** Total length 3.64. Carapace 1.46 long, 1.29 wide. Abdomen 2.29 long, 0.93 wide. ***Eye sizes and interdistances***: AME 0.05, ALE 0.05, PME 0.03, PLE 0.06, AME–AME 0.16, AME–ALE 0.10, PME–PME 0.23, PME–PLE 0.24, MOQL 0.21, MOQA 0.26, MOQP 0.29, CH 0.15. Sternum 0.87 long, 0.68 wide. ***Measurements of legs***: I 6.03 (1.76, 2.23, 1.56, 1.38, 0.66), II 7.61 (2.18, 1.38, 2.03, 1.78, 0.89), III 4.94 (1.6, 1.67, 1.08, 0.59), IV 5.94 (1.91, 1.95, 1.43, 0.65). Leg formula: II-I-IV-III. Cheliceral furrow with one promarginal tooth.

**Figure 20.**
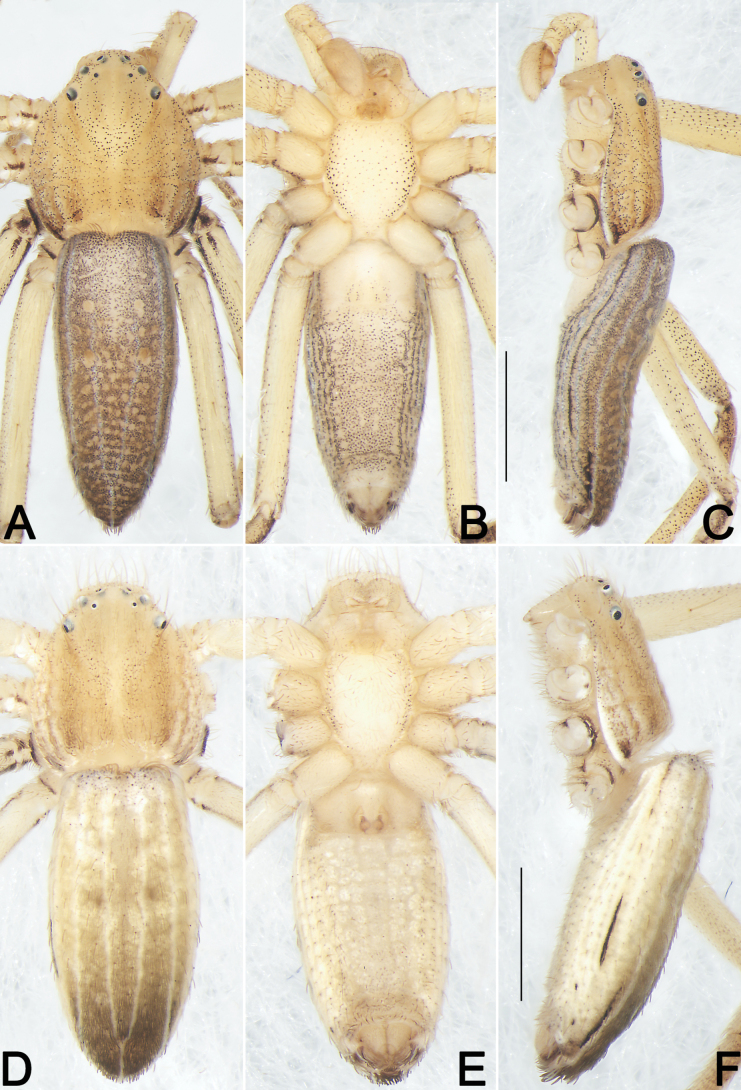
*Sinodromuslanyue* sp. nov., holotype male (**A–C**) and paratype female (**D–F**), habitus **A, D** dorsal view **B, E** ventral view **C, F** lateral view Scale bars: 1 mm (equal for **A–C**, equal for **D–F**).

***Colouration in ethanol*** (Figs 19F, 20A–C). Carapace basically yellow-brown, nearly pear-shaped, ocular region distinctly narrowed, tegument relatively smooth; with three pairs of indistinct, brown, longitudinal stripes, each one including dense black spots: the central pair starting from PLE, extending obliquely at front and vertically at rear, forming a funnel shape, or shaped like capital letter ‘Y’; the second pair also starting from PLE and extending almost vertically; the third pair running along the edge of the carapace, slightly curved, resembling a pair of parentheses. Chelicerae coloured slightly paler than carapace, cheliceral base with sparse black spots. Sternum uniformly yellowish-white, laterally with many black dots. Endites and labium coloured as cheliceral base, both with dense scopulae on anterior margins. All legs proximally yellowish-white (coxae, trochanters, and femora), distally brown (patellae, tibiae, metatarsi, and tarsi), with many small black dots on dorsal and lateral surfaces, covered by short spines. Abdomen elongate-oval, dorsum brown, clothed with dense hairs and covered by countless black spots, with two pairs of longitudinal, white lines, reaching entire abdominal length: the central pair anteriorly long and widely separated, posteriorly short and convergent, shaped like a tuning fork; the lateral pair running along the edge of the abdomen, almost ascending parallel; ventral abdomen basically yellowish-white, marked with small dense black dots.

***Palp*** (Figs 16A, B, 17A, B, 18A–D). Tibia relatively long, ~ 2/3 of Cy length, with two apophyses arising distally from tibia: VPTA relatively short, ~ 1/6–1/5 tibia length, subtriangular and nearly erect in ventral view, distinctly curved and dorsally toward posterior part of Te in prolateral view; RTA bifurcated, with a membranous, thumb-like dRTA and a relatively sclerotised, dagger-like vRTA, vRTA relatively long, ~ 1/3 tibia length, twice longer than dRTA. Cy ~ 1.9 × longer than wide, basoretrolaterally with an indistinct CP. Te egg-shaped, ~ 1.55 × longer than wide, proximally slightly swollen, prolatero-apically slightly excavated to accommodate Em and Con. SD sinuate, originating at retrolatero-distal portion of Te, proximally aligning clockwise along the tegular retrolateral margin, medially forming a S-shaped SDL in ventral view, with its distal end hidden behind Te and covered by Con, ultimately entering EmB. Em distinctly simplified and small, ~ 1/4 Te length, slightly curved, spine-like; EmT sharply pointed and directed retrolatero-distally, terminating at ~ 1 o’clock position. Con weakly sclerotised, with moderate size, ~ 1/3 Te length, basally columnar and slightly torqued along its length, apex triangular and terminating at ~ 1 o’clock position, covers Em. TA heavily sclerotised, shaped like a cock’s head, with a hump-like basal apophysis directed anteriorly and a sharp, beak-like apex pointing retrolaterally.

**Female (MGNU-2025-PHISL002).** Total length 3.93. Carapace 1.45 long, 1.31 wide. Abdomen 2.66 long, 1.14 wide. ***Eye sizes and interdistances***: AME 0.05, ALE 0.05, PME 0.03, PLE 0.06, AME–AME 0.19, AME–ALE 0.11, PME–PME 0.28, PME–PLE 0.25, MOQL 0.21, MOQA 0.28, MOQP 0.34, CH 0.17. Sternum 0.88 long, 0.64 wide. ***Measurements of legs***: I 4.73 (1.36, 1.81, 1.16, 0.99, 0.57), II 5.37 (1.64, 1.93, 1.31, 1.14, 0.66), III 4.32 (1.4, 1.50, 0.92, 0.5), IV 5.21 (1.73, 1.76, 1.14, 0.58). Leg formula: II-IV-I-III. Cheliceral furrow with one promarginal tooth. Colouration in ethanol as in males, but body slightly paler (Figs 19G, 20D–F).

***Epigyne*** (Fig. 19A–E). Epigynal field slightly wider than long; anterior and lateral margins not rebordered, posterior margin delimited; CD and R obscured through epigynal plate in ventral view. A small, located at antero-lateral part of epigynal plate, divided by anterior keel of MS, represented by two C-shaped depressions; the two depressions separated by ~ five diameters. MS more or less U-shaped, or vase-shaped, broad; anterior keel slightly narrowed, ~ 2/5 epigyne width, with distinct edges and delimited to A; medial stem slightly widened, ~ 1/2 epigyne width; posterior base nearly as wide as anterior keel; both middle stem and posterior base with indistinct lateral rMS alongside with rSEF. SEF shaped like a pair of parentheses; anteriorly distinctly widened, forming axe-shaped ET; midsection narrowed, with distinct edges and delimited to A; posteriorly widened, rSEF not distinct and alongside with rMS. CO indistinct, located at antero-lateral borders of MS, leading to CD which looped to connect with R. CD relatively short, ~ 1/4 epigyne length, with a course forming one loop before entering R. R close together, oval, ~ 1.2 × longer than wide, ~ 1/2 epigyne length and 1/3 epigyne width; receptacular surface hyaline and smooth, inside pigmented, sclerotised and granular. GM distinctly small, slightly protruding, papilliform, located at the antero-lateral surfaces of R. FD membranous and acicular, moderately long, ~ 2/5 of R length, originating from the posterior surface of R, directing antero-laterally.

##### Distribution.

Known from the type locality in Hubei Province, China (Fig. 23B).

#### ﻿Genus Tibellus

Taxon classificationAnimaliaAraneaePhilodromidae

Simon, 1875

6BA9B40A-88D7-506F-AA92-6CD4C5DD202A

##### Type species.

*Araneaoblonga* Walckenaer, 1802 from North America, Europe, North Africa, Turkey, Israel, Caucasus, Russia (Europe to Far East), Kazakhstan, Iran, Central Asia, Mongolia, China, Korea, Japan.

##### Diagnosis.

See Van den Berg and Dippenaar-Schoeman (1994) and Efimik (1999).

##### Comments.

The genus has been widely considered as putatively monophyletic; it presents a distinct set of characters (Van den Berg and Dippenaar-Schoeman 1994; Efimik 1999; Jang et al. 2023), and its species composition is relatively stable (WSC 2025). However, some species have extraordinarily intraspecific morphological variation and low levels of interspecific variation, and together with the insufficiency of alpha taxonomic information (lacking high-quality illustrations, detailed descriptions, and molecular data), have hindered species recognition and has resulted in several wrong descriptions and misidentifications (Efimik 1999).

#### ﻿ Tibellus japonicus

Taxon classificationAnimaliaAraneaePhilodromidae

Efimik, 1999

FFF24DD0-02B4-5D6D-BEC9-99D53444FF12

**Figure 21.**
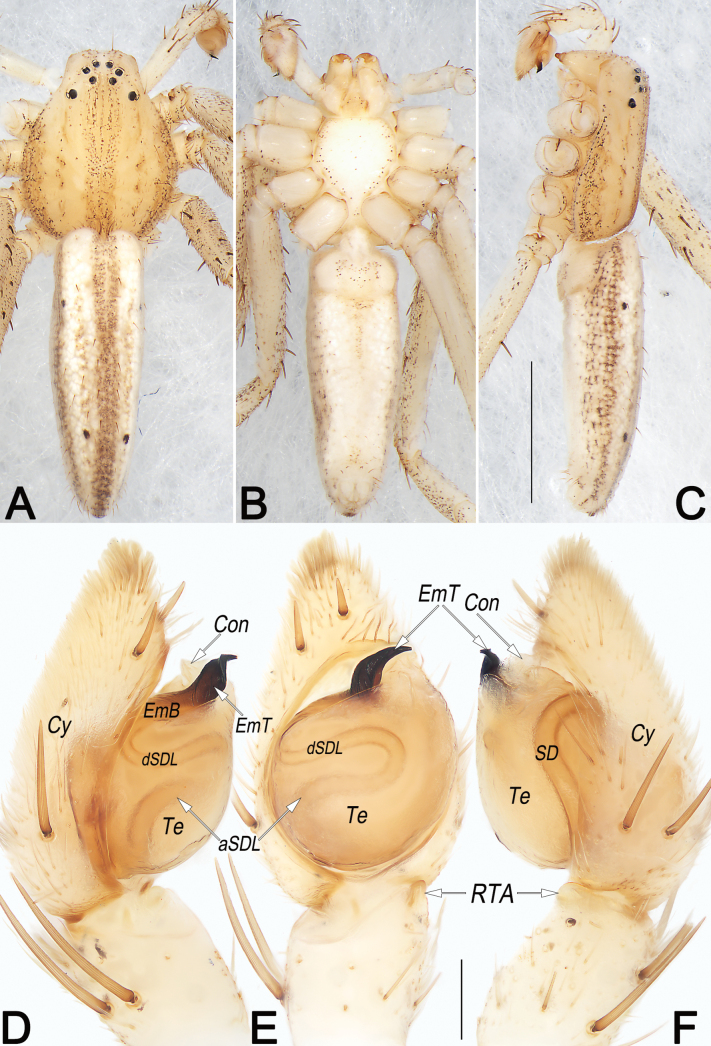
*Tibellusjaponicus*, male, habitus (**A–C**) and left palp (**D–F**) **A** dorsal view **B** ventral view **C** lateral view **D** prolateral view **E** ventral view **F** retrolateral view. Abbreviations: aSDL = ascending part of sperm duct loop; Con = conductor; Cy = cymbium; dSDL = descending part of sperm duct loop; EmB = embolic base; EmT = embolic tip; RTA = retrolateral tibial apophysis; SD = sperm duct; Te = tegulum. Scale bars: 1 mm (equal for **A–C**); 0.2 mm (equal for **D–F**).

**Figure 22.**
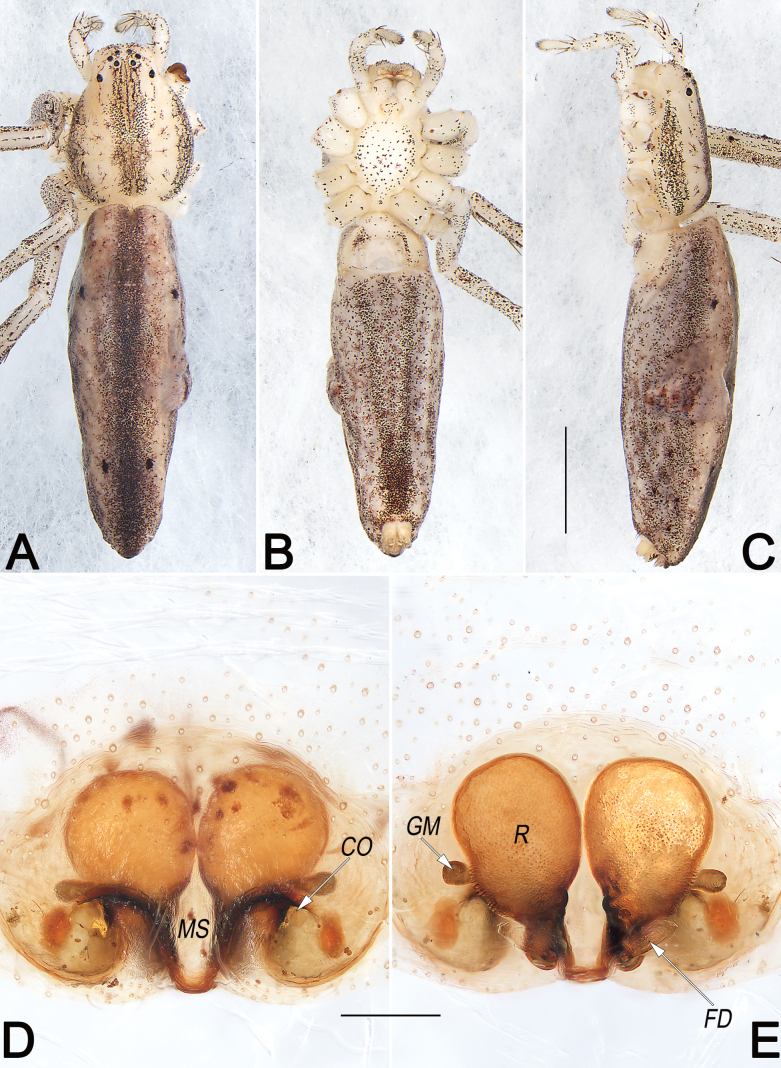
*Tibellusjaponicus*, female, habitus (**A–C**) and epigyne (**D, E**) **A** dorsal view **B** ventral view **C** lateral view **D** ventral view **E** dorsal view. Abbreviations: CO = copulatory opening; FD = fertilisation duct; GM = glandular mound; MS = median septum; R = receptaculum. Scale bars: 2 mm (equal for **A–C**); 0.2 mm (equal for **D, E**).

**Figure 23.**
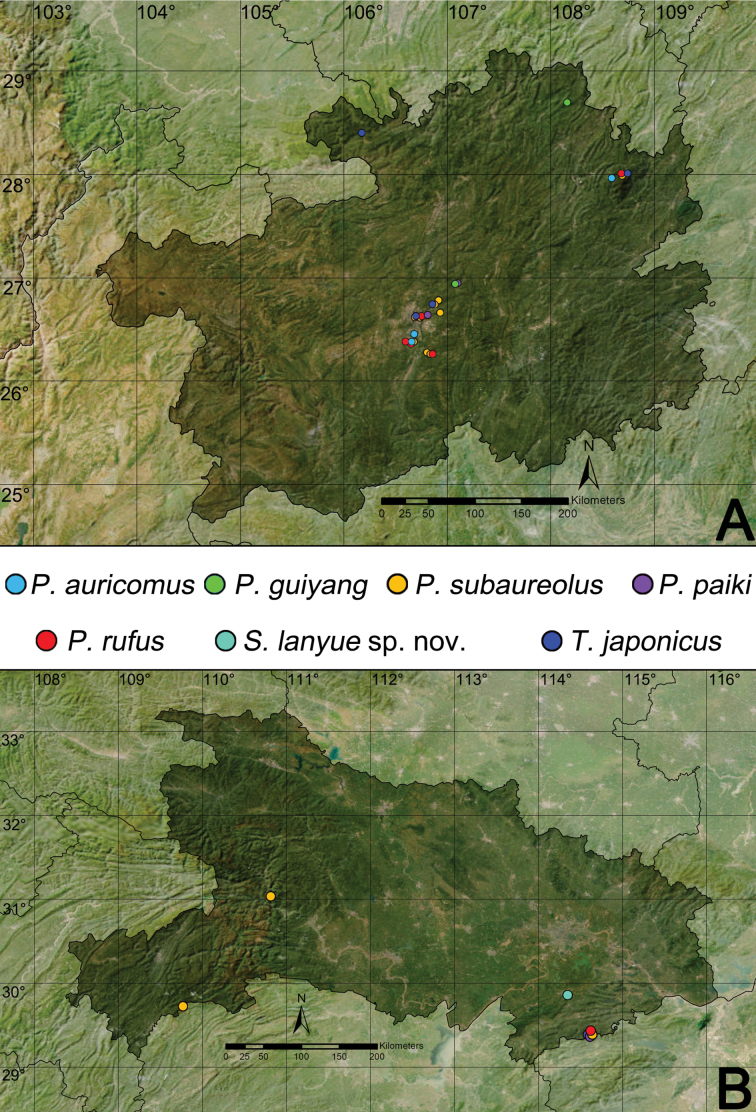
Distribution records of the philodromid species in Guizhou Province (**A**) and Hubei Province (**B**).

Figs 1 , 4C, D , 21 , 22 , 23

Tibellus tenellus : Bösenberg and Strand 1906: 271, pl. 8, fig. 112, pl. 10, fig. 156 (♂♀; misidentified per Efimik 1999: 112); Chikuni 1989: 133, fig. 2 (♂♀; misidentified per Efimik 1999: 112).
Tibellus japonicus Efimik, 1999: 112, figs 35, 46, 52, 65 (♀); Chen et al. 2003: 91, figs 1–5 (♂♀); Ono and Ban 2009: 478, figs 24–27 (♂♀); Yin et al. 2012: 1252, fig. 673a–d (♂♀); Jang et al. 2023: 278, fig. 4A–H (♂).^4^

##### Material examined.

**China**: Guizhou Province: • 1♂, 1♀ (YHGY301 and YHGY303 used for sequencing, GenBank accession numbers in Table 1), Guiyang City, Yunyan District, Luchongguan Forest Park; 26.63°N, 106.70°E; 1310 m a.s.l.; 4 June 2022; H. Yu et al. leg. • 1♀ (YHGY014 used for sequencing, GenBank accession numbers in Table 1), Guiyang City, Wudang District, Panlongshan Forest Park; 26.74°N, 106.88°E; 1172 m a.s.l.; 2 June 2022; H. Yu et al. leg. • 1♂, 1♀, Tongren City, Fanjingshan National Nature Reserve, Yinjiang Tujia and Miao Autonomous County, Tianqing Temple; 28.00°N, 108.74°E; 1462 m a.s.l.; 21 July 2021; H. Yu et al. leg. • 1♀, Zunyi City, Xishui National Nature Reserve, Xishui County, Donghuang Town, Changqiangou Village; 28.40°N, 106.18°E; 981 m a.s.l.; 28 May 2022; H. Yu et al. leg.; Hubei Province: • 1♂, 1♀, Xianning City, Jiugongshan National Nature Reserve, Yunzhonghu Scenic Area; 29.39°N, 114.65°E; 480 m a.s.l.; 3 July 2020; Q. Lu et al. leg.

##### Diagnosis and description.

See Efimik (1999), Chen et al. (2003), Yin et al. (2012), and Jang et al. (2023). Habitus as in Figs 21A–C, 22A–C. Male palp as in Fig. 21 D–F, epigyne as in Fig. 22D, E.

##### Distribution.

Russia (Southern Sakhalin), China (Guizhou, Hubei; Fig. 23), Korea, Japan.

##### Comments.

The species was reported as a new record for China by Chen et al. (2003) based on materials from Xishui National Nature Reserve in Guizhou. Subsequent literature has reported the distribution of this species in Henan and Hunan provinces (Zhu and Zhang 2011; Yin et al. 2012). Newly available specimens indicate that the species is also distributed in Guiyang City and Fanjingshan National Nature Reserve in Guizhou Province (Fig. 23A), as well as Jiugongshan National Nature Reserve in Hubei Province (Fig. 23B). This paper provides the first formal report of the species’ distribution in Hubei Province.

## ﻿Conclusions

This study presents the most comprehensive taxonomic account to date of philodromid spiders from Guizhou and Hubei provinces, China. A total of three genera and seven species are recorded, including the description of one new species, *Sinodromuslanyue* sp. nov., and several newly documented provincial records. Detailed morphological examinations, supplemented by DNA barcoding, have allowed for the clarification of previous misidentifications, the expansion of known species distributions, and the first English description of the male of *Philodromusguiyang*. These results reveal that the philodromid fauna in central and southwestern China remains insufficiently explored. Continued fieldwork and integrative taxonomic research across poorly studied regions of China will be essential for improving our understanding of philodromid diversity, and for advancing future phylogenetic and biogeographic studies of this widely distributed but understudied spider family.

## Supplementary Material

XML Treatment for Philodromus

XML Treatment for Philodromus auricomus

XML Treatment for Philodromus guiyang

XML Treatment for Philodromus subaureolus

XML Treatment for Philodromus paiki

XML Treatment for Philodromus rufus

XML Treatment for Sinodromus

XML Treatment for Sinodromus lanyue

XML Treatment for Tibellus

XML Treatment for Tibellus japonicus

## Footnotes

For full list of taxonomic references, see WSC (2025).

For full list of taxonomic references see WSC (2025).

For full list of taxonomic references, see WSC (2025).

For full list of taxonomic references, see WSC (2025).

## ﻿Additional information

### Author ORCIDs

Jianshuang Zhang https://orcid.org/0000-0003-4010-3082

Chengwen Zhang https://orcid.org/0009-0008-1258-5727

Yang Zhong https://orcid.org/0000-0002-0517-4582

### Data availability

All of the data that support the findings of this study are available in the main text.
